# Lactoferrin attenuates cardiac fibrosis and cardiac remodeling after myocardial infarction via inhibiting mTORC1/S6K signaling pathway

**DOI:** 10.7150/thno.85361

**Published:** 2023-06-04

**Authors:** Tianbao Ye, Zhiwen Yan, Cheng Chen, Di Wang, Aiting Wang, Taixi Li, Boshen Yang, Xianting Ding, Chengxing Shen

**Affiliations:** 1 Department of Cardiology, Shanghai Sixth People's Hospital Affiliated to Shanghai Jiao Tong University School of Medicine, Shanghai 200233, China.; 2 Institute for Personalized Medicine, School of Biomedical Engineering, Shanghai Jiao Tong University, Shanghai 200030, China.; 3 Youth Science and Technology Innovation Studio of Shanghai Jiao Tong University School of Medicine, Shanghai 200233, China.; 4 School of Medicine, Tongji University, Shanghai 200092, China.

**Keywords:** 'cardiac fibrosis', 'myocardial infarction', 'myofibroblast', 'lactoferrin', 'cardiac remodeling'

## Abstract

**Rationale:** Myocardial infarction (MI) causes a severe injury response that eventually leads to adverse cardiac remodeling and heart failure. Lactoferrin (Ltf), as a secreted protein, bears multi-pharmacological properties. Present study aims to establish the cardioprotective function and corresponding mechanism of Ltf in MI process.

**Methods and results:** We performed proteomic analysis in Tregs derived from MI heart, and identified Ltf as a remarkably upregulated secreted protein. However, Ltf was decreased in circulation and positively correlated with cardiac function both in mice and patients after MI. Ltf administration remarkably alleviated cardiac fibrosis and remodeling, improved cardiac function, and reduced incidence of heart failure in mice post-MI. *In vitro*, Ltf suppressed fibroblast to myofibroblast conversion induced by transforming growth factor-β (TGF-β). Mechanistically, phosphoproteomic landscape analysis revealed that Ltf repressed the activation of mTORC1/S6K/eIF-4B signaling pathway via interaction with CD74 receptor. Administration of mTORC1/S6K/eIF-4B axis agonist MHY1485 abolished the cardioprotective effects of Ltf. Besides, MHY1485 also markedly reversed the effects of Ltf on suppressing the transformation of fibroblast to myofibroblast mediated by TGF-β.

**Conclusion:** Our study established the cardiac protective role of Ltf in attenuating cardiac remodeling and improving cardiac function by inhibiting the activation of myofibroblasts through suppressing mTORC1/S6K/eIF-4B signaling pathway post-MI. Treatment with Ltf may serve as a potential novel therapeutic intervention in patients with MI.

## Introduction

Myocardial infarction (MI) is characterized with high morbidity and mortality worldwide 1. Due to the limited regeneration capacity of adult cardiomyocytes, it is important to form stable scar immediately post-MI for stabilizing and reinforcing the left ventricle (LV) to prevent heart rupture 2-4. Cardiac fibrosis, defined by excessive collagen-dominated extracellular matrix deposition, occurs in most chronic myocardial conditions and critically determines pathological remodeling including the integrity and physiology of heart, which in turn may lead to heart failure 2,4,5. Furthermore, increased mechanical strain in border area induced by collagen-rich scar may result in expansion of fibrosis area, decline of tissue compliance, and increase of cardiac afterload 6. Cardiac fibroblasts, as the primary profibrogenic cell population, could switch to myofibroblasts with enhanced proliferative, hypersecreted and hypercontractile capacities under injurious stimuli 3,4. Therefore, targeting myofibroblasts serves as a desirable therapeutic avenue to limit fibrotic overactivity.

MI stimuli instigates excessive and prolonged inflammatory process, leading to tissue injury, adverse remodeling and impaired cardiac function. Regulatory T-cells (Tregs) have been demonstrated as a key orchestrator in maintaining immune homeostasis and regulating inflammatory response in many diseases 7,8. Recent studies have shown that Tregs accumulating in heart after MI can regulate local inflammation, macrophage differentiation and myofibroblasts activation 8-12. Elimination of the Tregs recruited to heart exacerbates cardiac injury post-MI, while expansion of Tregs can protect heart from ischemic injury 9,11-13. Hence, it is widely recognized that Tregs derived secreted factors play a major protective role in MI injury.

Lactoferrin (Ltf), known as an iron-binding glycoprotein, is the most abundant element in milk 14. Prior research has illustrated that Ltf bears multi-pharmacological properties, including protection against infection, regulation inflammatory response, antioxidant and antifibrotic process 15-18. Moreover, clinical trials have identified the safety of oral administration of bovine Ltf, and demonstrated the efficacy of Ltf on anti-infection, treatment of inflammation, cancer, metabolic disorders 17,19-21. However, the potential role and underlying mechanism of Ltf on MI remain elusive.

In the current study, we performed a label-free proteomic analysis and identified Ltf as a critical upregulated secreted factor in Tregs after MI. We presented the first direct evidence that Ltf could attenuate adverse cardiac remodeling and improve cardiac function via inhibiting excessive cardiac fibrosis post-MI, mainly through suppressing the activation of mTORC1/S6K/eIF-4B axis in myofibroblasts. Additionally, we demonstrated that Ltf could directly interact with CD74 to repress the mTORC1/S6K/eIF-4B signaling pathway. Our study highlights a novel function of Ltf on suppressing detrimental cardiac remodeling post-MI stimuli, and hints that Ltf may be used to improve outcomes in patients with MI.

## Results

### Integrative proteomic analyses present Ltf as a key factor to promote cardiac repair after MI

**Figure 1:**
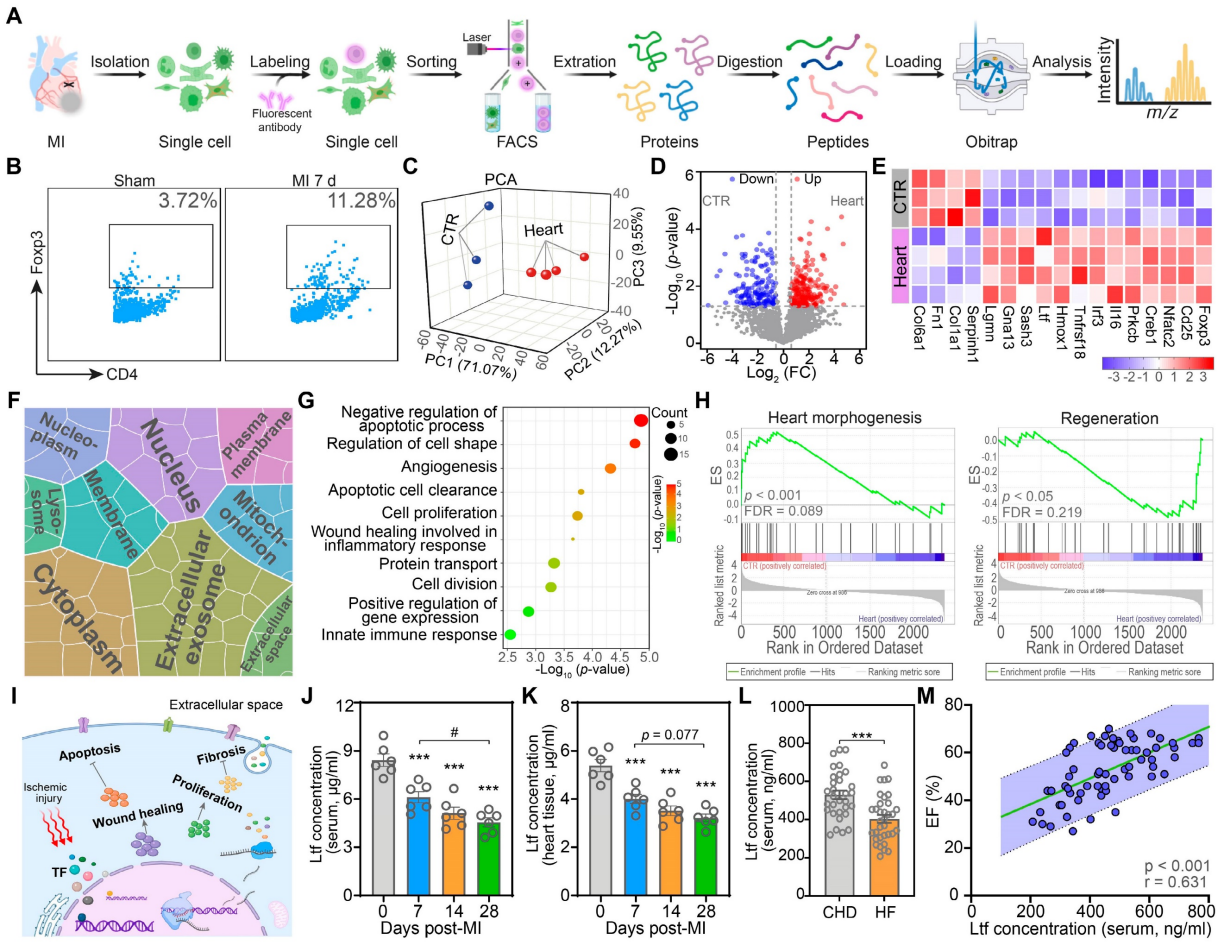
**Proteomic analyses present lactoferrin as a key factor to promote cardiac repair after MI. A**. Schematic of proteomics for Tregs. **B**. Representative flow cytometry analysis of cardiac Tregs post-MI, gating in CD45^+^CD3^+^CD4^+^ population. Numbers serve as the proportion of cells in the frame. **C**. PCA analysis of the proteomic profiling of naïve Tregs and heart Tregs. Each plot represents one biological replicate. **D**. Volcano plots analysis of proteins comparing between naïve Tregs and heart Tregs. **E**. Heat map of selected list of DEPs. **F**. Cell component analysis of DEPs. Area represents enrichment. **G**. Biological process terms of DEPs. **H**. GSEA analysis via GO biological process database. Adjusted p-value and FDR are shown for each enrichment dataset. **I**. Proposed model of Tregs-triggering cardiac protective function after MI. **J-L**. The Ltf protein level in mice serum (**J**, n = 6/ea), supernatant of mice cardiac homogenate (**K**, n = 6/ea), and serum derived from patients (**L**, n = 31-34/ea). **M**. Correlation analysis of circulating Ltf concentration and EF in patients. *p*-value and correlation coefficient are presented. n = 65. Data are presented as mean ± SEM. **J** and** K**, by one-way ANOVA followed by Bonferroni post hoc test; **L**, by unpaired student* t* test; **M**, by Pearson correlation test. ***P < 0.001 compared with control group, ^#^P < 0.05 compared with the indicated group.

Previous studies confirm that Tregs exert protective effects post-MI via alleviating local inflammation, promoting wound healing, reducing cardiomyocytes apoptosis, and modulating myofibroblasts activation 8,9. To gain more profound insights into the action of cardiac Tregs, we performed proteomic experiments on cardiac Tregs in mice at day 7 post-MI to assess the alteration of protein profiling (**Figure 1A**). Due to the rarity of cardiac resident Tregs in basic state and the recruitment of cardiac Tregs after MI mainly derived from secondary lymphoid tissue, spleen derived naïve Tregs under sham procedure were served as control (CTR) 8,13. Consistent with previous studies 8,10, Tregs in myocardium were increased significantly at day 7 post-MI, while the change in spleen was not obvious (**Figure 1B**; **Figure S1**). Next, principal component analysis (PCA) demonstrated proteomic difference between naïve Tregs and cardiac Tregs (**Figure 1C**). Volcano plots revealed that 209 proteins were upregulated while 153 proteins were downregulated in cardiac Tregs compared with naïve Tregs (**Figure 1D**). Among the differentially expressed proteins (DEPs), canonical Treg signatures (Foxp3, CD25), cytokines (Il-16, Irf3), and proliferation-associated proteins (Homx1, Gna13, Ltf) were upregulated, but the extracellular matrix and collagen-related proteins were decreased in cardiac Tregs (**Figure 1E**).

To identify the functionality and subcellular localization of the DEPs, gene ontology (GO) analysis was conducted. The cell component (CC) analysis revealed that DEPs were enriched in extracellular environment, cytoplasm and nucleus, suggesting active protein synthesis and secreting processes in cardiac Tregs (**Figure 1F**). Furthermore, biological process (BP) terms of cardiac Tregs were mainly related to anti-apoptosis, angiogenesis, cell proliferation, and wound healing (**Figure 1G**). In addition, Gene set enrichment analysis (GSEA) showed that naïve Tregs exerted their main effect on heart morphology and development, while cardiac Tregs were primarily involved in the repair process after MI injury (**Figure 1H**; **Figure S2**). Taken together, our data suggest cardiac Tregs present enhanced capacity of protein-synthesis and secreting, which are potentially involved in cardiac repair after MI via promoting cardiomyocytes survival, angiogenesis, wound healing, and inhibiting cardiac fibrosis (**Figure 1I**).

Ltf, as a secreted protein, was highly expressed in cardiac Tregs according to the proteomics analysis. Consistent with the mass spectrometric experiment, qRT-PCR results showed a significant increase of *Ltf* expression in cardiac Tregs compared with naïve Tregs (**Figure S3A**). By contrast, the Ltf protein level in serum and myocardium were markedly decreased with disease progression in mice after MI injury (**Figure 1J-K**; **Figure S3B**). Coincidently, Ltf protein level was also decreased in serum derived from MI patients with heart failure (**Figure 1L**; **Table S1**). What's more, both the serum level of Ltf in mice and patients presented a positive correlation with cardiac function after MI (**Figure 1M**; **Figure S3C**). Previous studies have reported that Ltf can promote CD4+ T cells skewing towards a Treg population and then migrating to injured areas for tissue repair 22. Our flowcytometry assays showed that exogenous Ltf supplementation increased Tregs population in heart after MI (**Figure S3D**). Integrating what was discussed above, we proposed the hypothesis that exogenous Ltf supplementation could improve cardiac function after MI.

### Ltf alleviates adverse ventricular remodeling and improves cardiac function after MI

**Figure 2:**
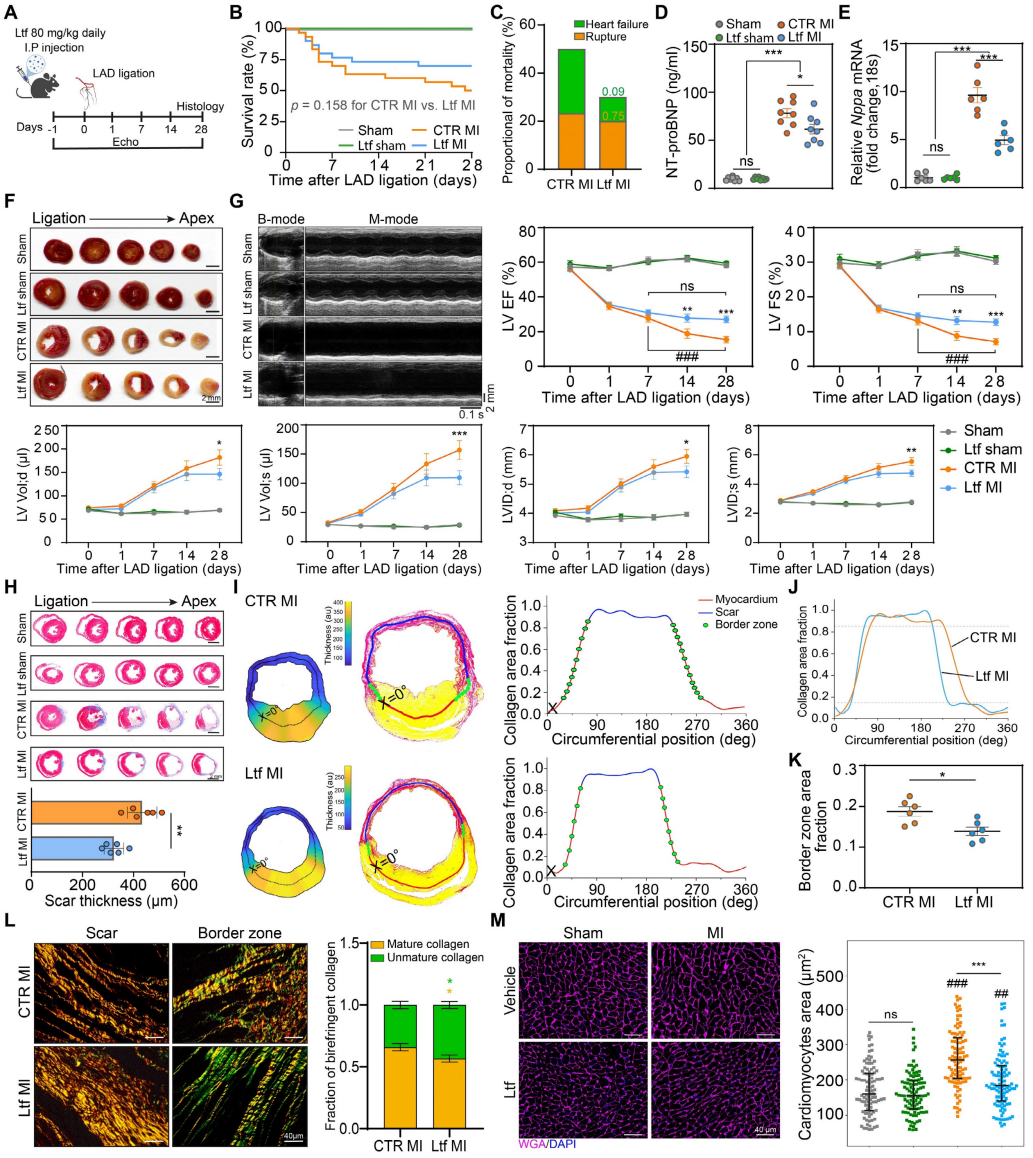
**Ltf alleviates adverse ventricular remodeling and improves cardiac function after MI. A**. Diagram of the experimental approach. **B**. Survival curve of the corresponding subgroups. **C**. Proportion of cardiac rupture and heart failure causing death among mice in indicated MI groups. Numbers serve as the *p*-values. **D**. NT-proBNP level in serum. n = 8/ea. **E**. mRNA expression level of *Nppa* in myocardium. n = 6/ea. **F**. Representative images of TTC staining at day 1 post-MI. **G**. Representative serial echocardiography via long-axis acquired at different timepoints and relative quantifications. n = 10-15/ea. **H**. Five serial sections of representative Masson trichrome staining (up). Quantification of scar thickness is shown (down). n = 6/ea. **I**. Analytic method of calculating scar thickness visualized with pseudo-colored image (left), demarcation of scar, border zone and remote zone (middle), and the definition of border zone (right) via a custom-built image processing pipeline. **J**. Representative curve of collagen area fraction. **K**. Quantification of border zone transition rate according to the pipeline analysis. n = 6/ea. **L**. Picrosirius red staining imaged under polarized light in the scar area (left) and border zone (right) followed with calculating frequency of birefringent collagen (red/yellow presents mature collagen and green presents unmature collagen). n = 6/ea. **M**. Representative images of WGA staining of cardiomyocytes 28 day after MI. Quantification is shown on the right. n = 5-6/ea. Data are presented as mean ± SEM. **B**, by Kaplan-Meier survival test; **C**, by Chi-square test; **D** and **E**, by one-way ANOVA followed by Bonferroni post hoc test; **G**, by two-way ANOVA followed by Bonferroni post hoc test; **H**, **K** and **L**, by unpaired student* t* test; **M**, by Mann-Whitney non-parametric test; *P < 0.05, **P < 0.001, ***P < 0.001 compared with the corresponding group;^ ##^P < 0.01,^ ###^P < 0.001 compared with corresponding sham group. **G**, *P < 0.05, **P < 0.001, ***P < 0.001 compared with CTR MI group; ^###^P < 0.001 compared with day 7 in CTR MI group. Ltf MI, mice treated with Ltf under MI operation; CTR MI, mice treated with vehicle under MI operation.

In order to test our hypothesis that exogenous supplementation of Ltf might stimulate cardiac repair after MI, we conducted a series of *in vivo* experiments. Ltf was applied intraperitoneally daily from surgery to 4 weeks post-MI (**Figure 2A**). After administration of Ltf, circulating Ltf maintained a high level over the whole study period (**Figure S4A**). Exogenous supplementation of Ltf presented an increasing trend of survival rate compared to CTR MI group, with a decreased proportional death of heart failure (**Figure 2B-C**; **Figure S4B**). Additionally, heart failure indicators (NT-proBNP, *Nppa* and *Nppb*) were significantly decreased in Ltf administration MI group (**Figure 2D-E**; **Figure S4C**). Meanwhile, lower heart weight/tibia length ratio and lung wet/dry ratio were observed after Ltf treatment (**Figure S4D-E**). These data suggest that Ltf can reduce incidence of heart failure after MI injury.

Although Ltf administration did not affect the infarct size and severity of myocardial injury (**Figure 2F**; **Figure S4F-G**), treatment with Ltf significantly improved cardiac function and attenuated cardiac remodeling, as presented with significantly higher ejection fraction, fractional shortening and smaller left ventricular dimension post-MI (**Figure 2G**; **Figure S5** and **Table S2**). Consistent with the TTC assay, Masson trichrome-stained analysis revealed that supplementation of Ltf did not affect infarct size (**Figure 2H**; **Figure S6A**). While the scar in Ltf-treated group appeared to be thinner, indicating diminished collagen deposition (**Figure 2H**). Furthermore, via a custom-built image processing pipeline, scar thickness, infarct size and border zone area were calculated accurately with picrosirius red staining 3. In accordance with Masson trichrome-stained results, Ltf treated mice presented a thinner scar, instead of equal infarct size (**Figure 2I-J**; **Figure S6B-C**). Besides, administration of Ltf resulted in a smaller expansion of border zone with lower rate of border zone transition (**Figure 2K**). The area fraction of collagen in the remote zone was similar with sham groups (**Figure S6D-E**). These findings suggest that Ltf treatment remarkably depresses fibrosis in injured regions after MI.

Because difference in fibrotic response was obviously observed, polarized light was used to further analyze the picrosirius red stained samples. Well-aligned fiber in the scar area was observed assistance with Ltf treatment, but collagen fiber thickness was not affected (**Figure 2L**). Besides, a higher proportion of less mature collagen (green) was seen in border zone in mice treated with Ltf (**Figure 2L**). Increased extracellular matrix collagen deposition can result in tissue stiffness, which in turn contributes to a hypertrophic response in cardiomyocytes 4. Wheat germ agglutinin (WGA) staining was performed to define cardiomyocytes hypertrophy at 4 weeks post-MI. The cardiomyocytes area was significantly increased in mice without Ltf treatment (**Figure 2M**). These results indicate Ltf can attenuate adverse ventricular remodeling to improve cardiac function post-MI in mice.

### Ltf regulates collagen remodeling after MI

**Figure 3:**
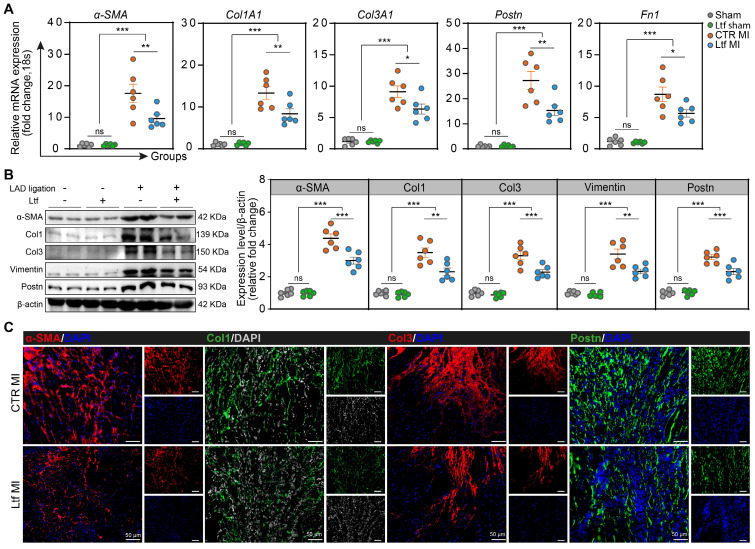
**Ltf regulates collagen remodeling after MI. A**. Relative mRNA expression of collagen-related genes and fibrosis-associated genes. n = 6/ea. **B**. Representative Western blot of indicated proteins in injured area at day 7 post-MI. n = 6/ea. **C**. Representative micrographs of immunofluorescence staining with indicated proteins in injured area at day 7 post-MI. Data are presented as mean ± SEM, by one-way ANOVA followed by Bonferroni post hoc test; *P < 0.05, **P < 0.001, ***P < 0.001 compared with corresponding group.

We next examined the potential effect of Ltf on collagen deposition after MI in mice. Supplementary treatment with Ltf resulted in reduced expression of collagen-related genes (*Col1A1* and *Col3A1*) and fibrosis-associated genes (*α-SMA*,* Postn* and *Fn1*) in the injured area at day 7 post-MI (**Figure 3A**). With regard to fibrosis-associated enzymes, the expression of *Lox* and *Timp1* tended to be lower, while *MMP-2* and *MMP-9* expression were not affected (**Figure S7A**). Western blot analysis confirmed that the α-SMA, Col1, Col3, Vimentin and Postn were remarkably downregulated in Ltf treated mice compared with CTR MI group at injured site (**Figure 3B**). Consistent with the qRT-PCR and Western blot assays, immunofluorescence staining also presented downregulated expression of α-SMA, Col1, Col3 and Postn after treatment with Ltf, but no changes were observed regarding to MMP2 and MMP9 (**Figure 3C**; **Figure S7B-D**). These results show that administration of Ltf exerts protective effects on inhibiting cardiac fibrosis to attenuate adverse remodeling after MI in mice.

Myocardial angiogenesis and cardiomyocytes apoptosis are tightly linked to cardiac remodeling after MI. We observed that microvascular in injured myocardium stained with CD31 was not significantly affected in MI mice supplemented with Ltf, suggesting that Ltf exerted negligible impact on angiogenesis capacity (**Figure S8A**). Simultaneously, more apoptotic cells were detected in the absence of Ltf supplementary treatment (**Figure S8B**). Together, administration of Ltf results in lower expression of collagen-related genes (*Col1A1* and* Col3A1*) and fibrosis-associated genes (*α-SMA*, *Postn* and *Fn1*), and reduces myocardial apoptosis post-MI which may contribute to attenuating cardiac fibrosis.

### Ltf suppresses TGF-β-induced fibroblast to myofibroblast conversion *in vitro*

**Figure 4:**
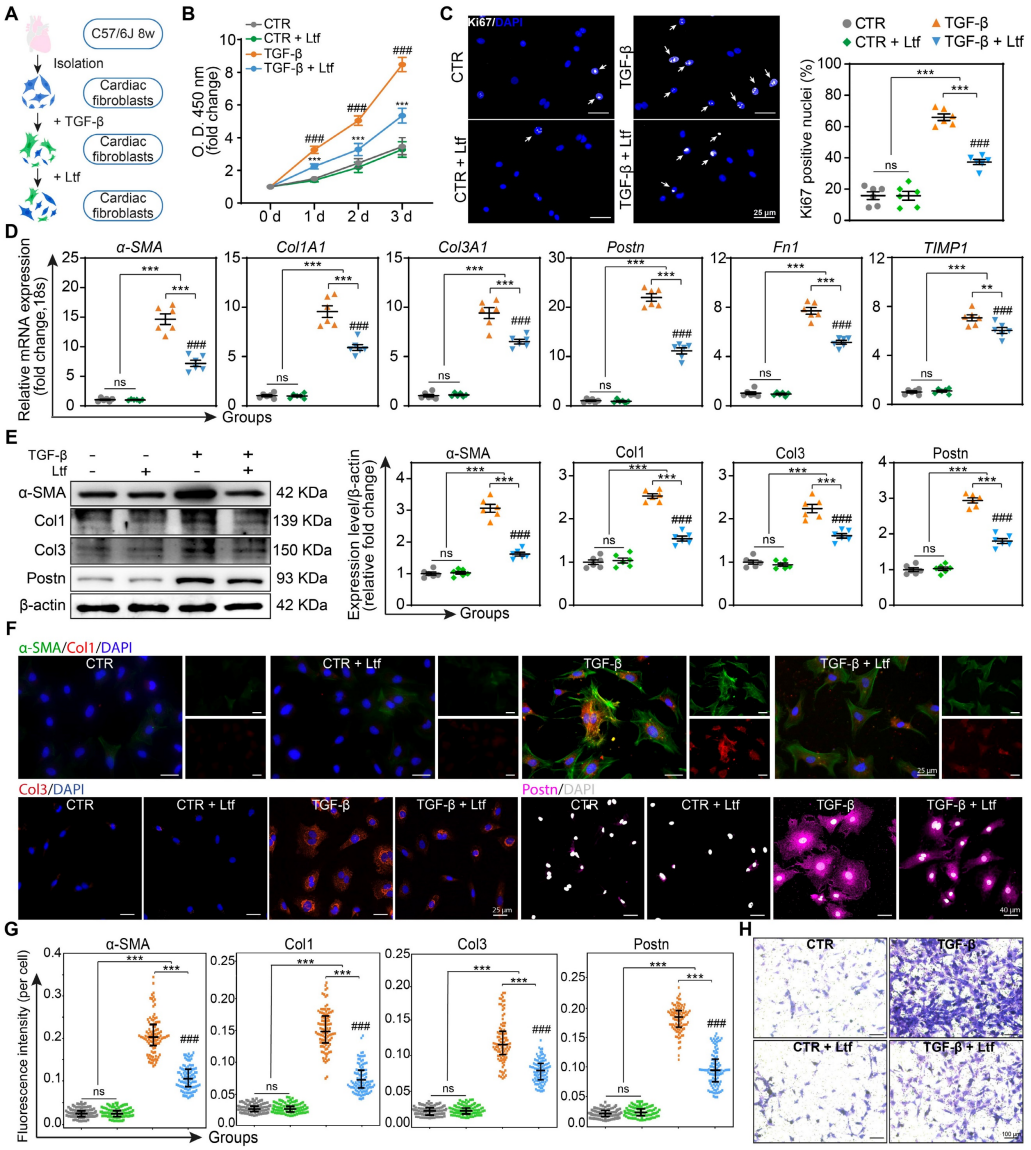
**Ltf suppresses TGF-β-induced fibroblast to myofibroblast conversion *in vitro*. A**. Schematic of the experimental protocol. **B**. Fibroblasts proliferation curves at indicated time. n = 8/ea. **C**. Representative immunofluorescent images of proliferative cells with Ki67-labeled nuclei. Quantification is shown on the right. n = 6/ea. **D**. Relative fold change of indicated mRNA expression level after stimulation. n = 6/ea. **E**. Representative Western blot of α-SMA, Col1, Col3 and Postn in cell lysates. n = 6/ea. **F**. Representative immunofluorescent micrographs of fibroblasts stained with indicated antibodies. **G**. Quantifications of fluorescence intensity. n = 6/ea. **H**. Representative images of transwell assay at 24 hours after stimulation. Data are presented as mean ± SEM. **B**, by two-way ANOVA followed by Bonferroni post hoc test; **C**, **D** and **E**, by one-way ANOVA followed by Bonferroni post hoc test; **G**, by Mann-Whitney non-parametric test, *P < 0.05, **P < 0.001, ***P < 0.001 compared with corresponding group. ^##^P < 0.01, ^###^P < 0.001 compared with corresponding control group. **B**, ***P < 0.001 compared with corresponding control group, ^###^P < 0.001 compared with Ltf group.

Transforming growth factor-beta (TGF-β), as the most effective mediator for fibroblast transformation into myofibroblast characterized with enhanced capacities of proliferation, collagen formation and migration, is rapidly elevated in the infarcted myocardium 23. Due to the obvious effect of Ltf on attenuating fibrosis remodeling after MI, we further investigated whether Ltf can reverse the activation of primary cardiac fibroblasts induced by TGF-β *in vitro* (**Figure 4A**). Administration of Ltf remarkably reduced the proliferation capacity of fibroblasts induced by TGF-β via the Cell Counting Kit-8 assay and Ki67 immunofluorescence staining (**Figure 4B-C**), indicating Ltf exerted beneficial roles in inhibiting proliferation of myofibroblasts. qRT-PCR assays demonstrated that TGF-β inducing upregulated expression of collagen-related genes (*Col1A1* and* Col3A1*), fibrosis-associated genes (*α-SMA*, *Postn* and* Fn1*) and fibrosis-associated enzymes (*Lox*, *Timp1*) were reversed after treatment with Ltf (**Figure 4D**; **Figure S9A**). Besides, TGF-β-mediated high expression of* MMP9* in fibroblasts was further augmented post Ltf supplementation (**Figure S9A**). Western blot analysis further confirmed α-SMA, Col1, Col3 and Postn were downregulated in fibroblasts treated with Ltf compared with TGF-β stimulation (**Figure 4E**). Additionally, immunofluorescent images also demonstrated TGF-β-mediated overexpression of α-SMA, Col1, Col3 and Postn were partially abolished by Ltf (**Figure 4F-G**). Furthermore, transwell assays showed that migratory capacity of fibroblasts was significantly inhibited by Ltf compared with TGF-β stimulation alone (**Figure 4H**; **Figure S9B**). These data illustrate that Ltf can effectively suppress the transformation of cardiac fibroblast to myofibroblast mediated by TGF-β.

### Ltf attenuates cardiac fibrosis via interacting With CD74 to inhibit mTORC1/S6K/eIF-4B signaling pathway

**Figure 5:**
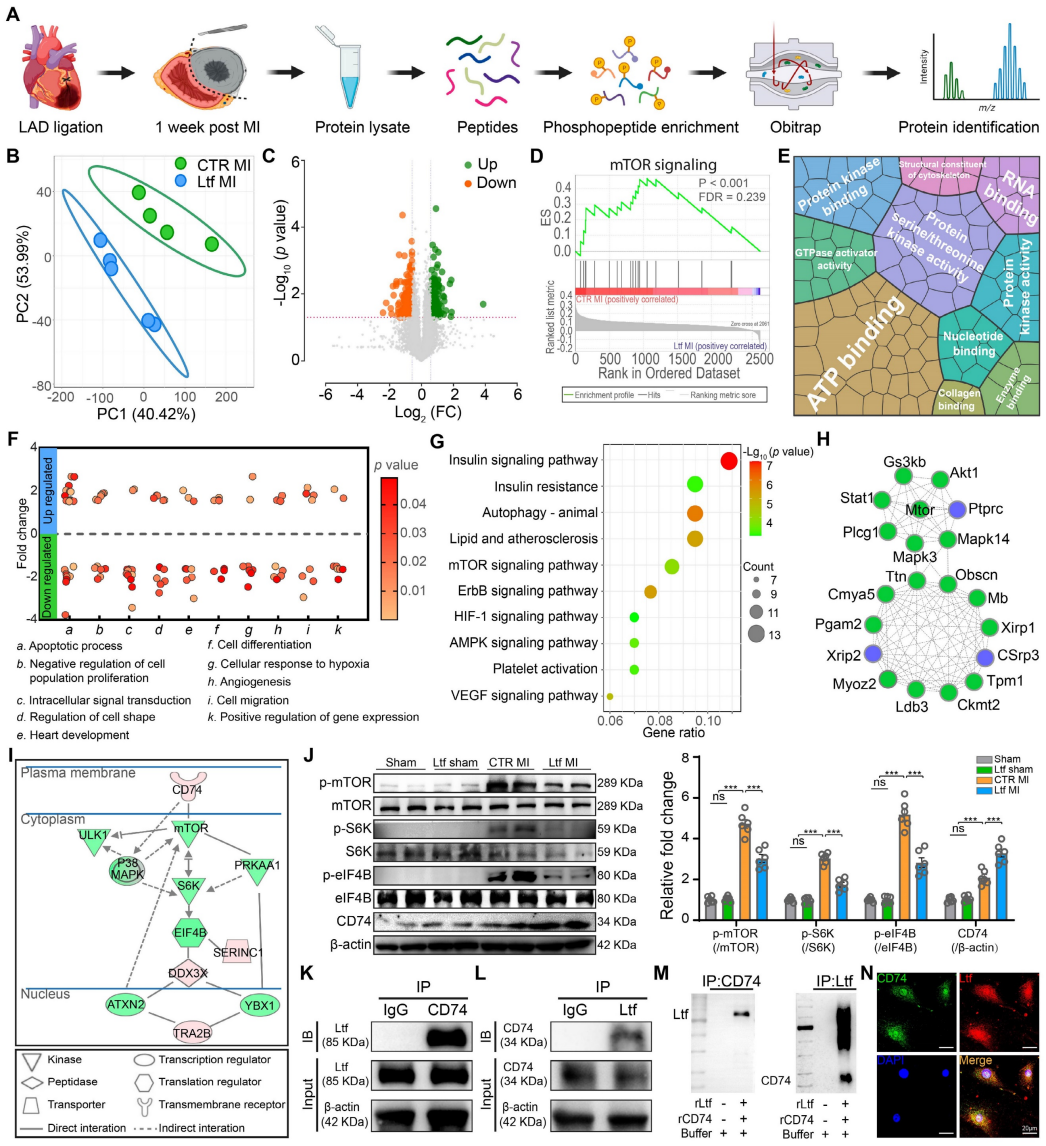
**Ltf attenuates cardiac fibrosis via interacting with CD74 to inhibit mTORC1/S6K/eIF-4B signaling pathway. A**. Schematic overview of phosphoproteomics. **B**. PLS-DA analysis of phosphoproteomic profiling between CTR MI and Ltf MI group. **C**. Volcano plots analysis of phosphopeptides. **D**. GSEA analysis via Reactome database. **E**. Enriched GO terms by molecular function. Area represents enrichments. **F**. Primary biological processes in the DMPs. The clusters are referred to biological process terms. Each dot represents a single DMP. **G**. Signaling pathway classification according to KEGG terms. **H**. Cytoscape analysis of protein-protein interactions network. The top 20 defined by cytoHubba were shown.** I**. Ingenuity pathway analysis of DMPs. **J**. Representative Western blot images (left) and quantitative analysis (right) of indicated proteins. n = 6/ea. **K**-**L**. Cellular co-immunoprecipitation assays of Ltf and CD74. Cell lysates were immunoprecipitated with antibody against CD74, immunoblot with Ltf antibody (**K**) and vice versa (**L**). **M**. Co-immunoprecipitation analysis of rLtf and rCD74 in a cell-free system. **N**. Immunofluorescence co-localization of Ltf and CD74 in fibroblasts. Data are presented as mean ± SEM, by one-way ANOVA followed by Bonferroni post hoc test, ***P < 0.001 compared with corresponding group.

To further investigate the molecular mechanism underlying the effect of Ltf on regulating cardiac fibrosis after MI, we performed a phosphoproteomics on infarcted mice hearts at day 7 post-MI (**Figure 5A**). About 11670 phosphorylated peptides attributing to 2828 quantifiable proteins were identified (**Figure S10A-B**). Partial least squares discrimination analysis (PLS-DA) demonstrated the different phosphorylation modification profiling between Ltf MI group and CTR MI group (**Figure 5B**). Volcano plots revealed that 161 phosphorylated sites were upregulated while 191 were downregulated in Ltf treated heart compared with control group (**Figure 5C**). To gain insight into the differently modified phosphoproteins (DMPs), we performed GSEA and GO analysis. GSEA analysis indicated mTOR signaling pathway was highly activated in CTR MI group (**Figure 5D**). Besides, term related to leukocyte transendothelial migration was also highly enriched in CTR MI group, suggesting more intense inflammation (**Figure S10C**). GO terms illustrated that DMPs exerted protein binding and kinase function, then participated in biological process with regard to apoptotic process, maintenance of cardiac morphology and angiogenesis, which were closely related to cardiac repair (**Figure 5E-F**; **Figure S10D**). These results confirm that Ltf exert cardioprotective function after MI via regulating the modification of cardiac repair-related proteins.

Next, pathway analysis was conducted to identify the primary pathways altered in mice treated with Ltf. KEGG analysis revealed insulin signaling pathway, autophagy related pathway, mTOR signaling pathway were highly enriched (**Figure 5G**). Protein-protein interaction network analysis demonstrated mTOR signaling pathway-associated proteins were highlighted in the cooperation network among the DMPs (**Figure 5H**). Additionally, ingenuity pathway analysis (IPA), which linked phosphoproteins with transcriptional regulators mediated by Ltf to predict specific functional pathway, showed that CD74 was predicted to be upstream in the mTORC pathway, and mTORC1/S6K/eIF-4B axis was predicted to be the primary signaling pathway related to Lft treatment (**Figure 5I**). Western blot showed that the elevated phosphorylated mTORC, S6K and eIF-4B induced by MI stimuli were suppressed after administration of Ltf *in vivo* (**Figure 5J**). Consistent with *in vivo* experiments, Ltf partially inhibited the activation of mTORC1/S6K/eIF-4B signaling pathway stimulated by TGF-β *in vitro*, instead of affecting mTORC2 pathway (**Figure S10E-G**). Taken together, these data indicate that mTORC1/S6K/eIF-4B axis serves as the primary pathway involved in Ltf-mediated suppression of cardiac fibrosis after MI.

It has been illustrated that CD74 can activate AMPK to exert cardioprotective function, and that AMPK can negatively inhibit the activity of mTORC1 24,25. Combined with IPA results, we supposed Ltf might interact with the receptor CD74, in turn suppressing the mTORC1/S6K/eIF-4B pathway. In fact, co-immunoprecipitation assays revealed an interaction between Ltf and CD74 in cell lysate derived from fibroblasts treated with Ltf (**Figure 5K-L**). Additionally, with co-incubation of recombinant Ltf and recombinant CD74 in a cell-free system, we determined Ltf directly interacted with CD74 as well (**Figure 5M**). Immunocytochemistry also presented colocalization of Ltf and CD74 in fibroblasts treated with Ltf (**Figure 5N**). Overall, our data suggest that Ltf can directly interact with CD74, leading to the suppression of the mTORC1/S6K/eIF-4B signaling pathway.

### Ltf represses TGF-β-induced fibroblast to myofibroblast transformation via inhibiting mTORC1/S6K/eIF-4B signaling pathway *in vitro*

**Figure 6:**
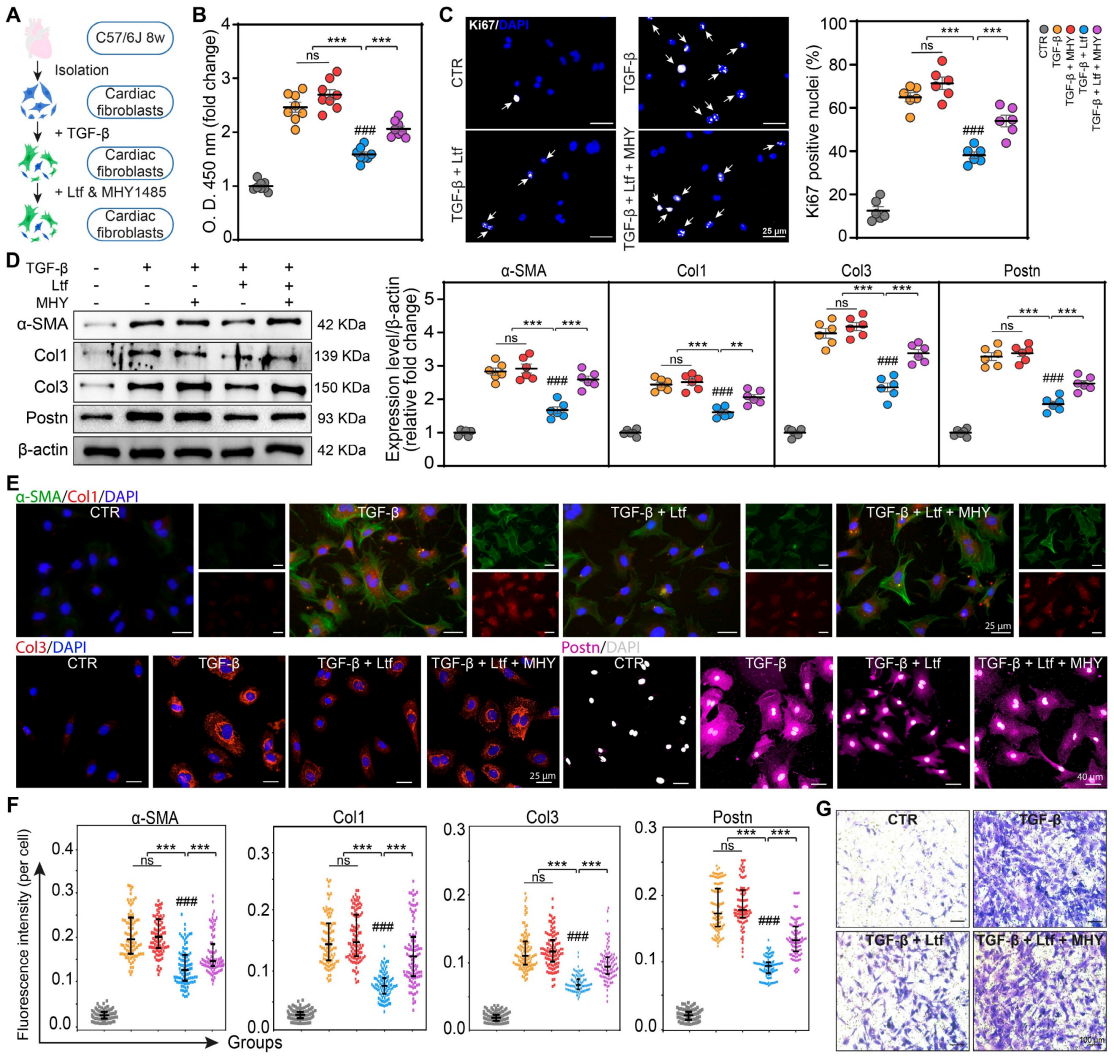
** Ltf represses TGF-β-induced fibroblast to myofibroblast transformation through inhibiting mTORC1/S6K/eIF-4B signaling pathway *in vitro*. A.** Schematic showing primary cardiac fibroblasts treated with TGF-β, Ltf and MHY1485. **B-C.** Proliferation assay of fibroblasts via CCK-8 assay (**B**, n = 8/ea) and immunofluorescent staining with Ki67 (**C**, n = 6/ea) 24 hours after treatment. **D.** Representative Western blot and quantifications of indicated proteins. n = 6/ea. **E-F.** Representative immunofluorescent micrographs (**E**) and quantification of fluorescence intensity (**F**) in fibroblasts stained with antibody against α-SMA, Col1, Col3 and Postn. n = 6/ea. **G.** Representative micrographs of transwell assay. Data are presented as mean ± SEM. **B**, **C** and **D**, by one-way ANOVA followed by Bonferroni post hoc test; **F**, by Mann-Whitney non-parametric test, *P < 0.05, **P < 0.001, ***P < 0.001 compared with corresponding group. ^###^P < 0.001 compared with control group.

We next tested whether the effect of Ltf on inhibiting fibroblast to myofibroblast conversion was primarily mediated through repressing mTORC1/S6K/eIF-4B axis. Recently, the activation of mTORC1/S6K/eIF-4B pathway by TGF-β has been confirmed 26-28, and MHY1485 as an effective agonist of mTORC1/S6K/eIF-4B pathway has been widely used 29,30. Primary cardiac fibroblasts were pretreated with TGF-β followed by Ltf and MHY1485 (**Figure 6A**). Ltf significantly restrained the mTORC1/S6K/eIF-4B axis activated by TGF-β in cardiac fibroblasts, while this effect was partially reversed by cotreatment with MHY1485 (**Figure S11**). Administration of MHY1485 significantly abolished the reduced proliferation capacity of myofibroblasts mediated by Ltf in the setting of TGF-β stimulation (**Figure 6B-C**; **Figure S12A**). Meanwhile, the downregulation of fibrosis-related genes (*Col1A1*, *Col3A1*, *α-SMA*, *Postn*, *Fn1* and *Lox*) under Ltf treatment were reversed after cotreatment with MHY1485 (**Figure S12B**). Subsequently, Western blot analysis demonstrated MHY1485 counteracted the effects of Ltf on repressing α-SMA, Col1, Col3 and Postn expression (**Figure 6D**). Consistent with Western blot results, immunofluorescent assays confirmed that the Ltf-induced downregulation of TGF-β-mediated α-SMA, Col1, Col3 and Postn expression was abolished after cotreatment with MHY1485 (**Figure 6-F**; **Figure S12C**). Migratory capacity of fibroblasts was significantly enhanced by cotreatment with MHY1485 compared with Ltf administration alone (**Figure 6G**; **Figure S12D**). These data further indicate that Ltf restrains TGF-β-induced fibroblast to myofibroblast conversion via suppressing mTORC1/S6K/eIF-4B signaling pathway.

### mTORC1 agonist reverses cardiac protection of Ltf after MI *in vivo*

**Figure 7:**
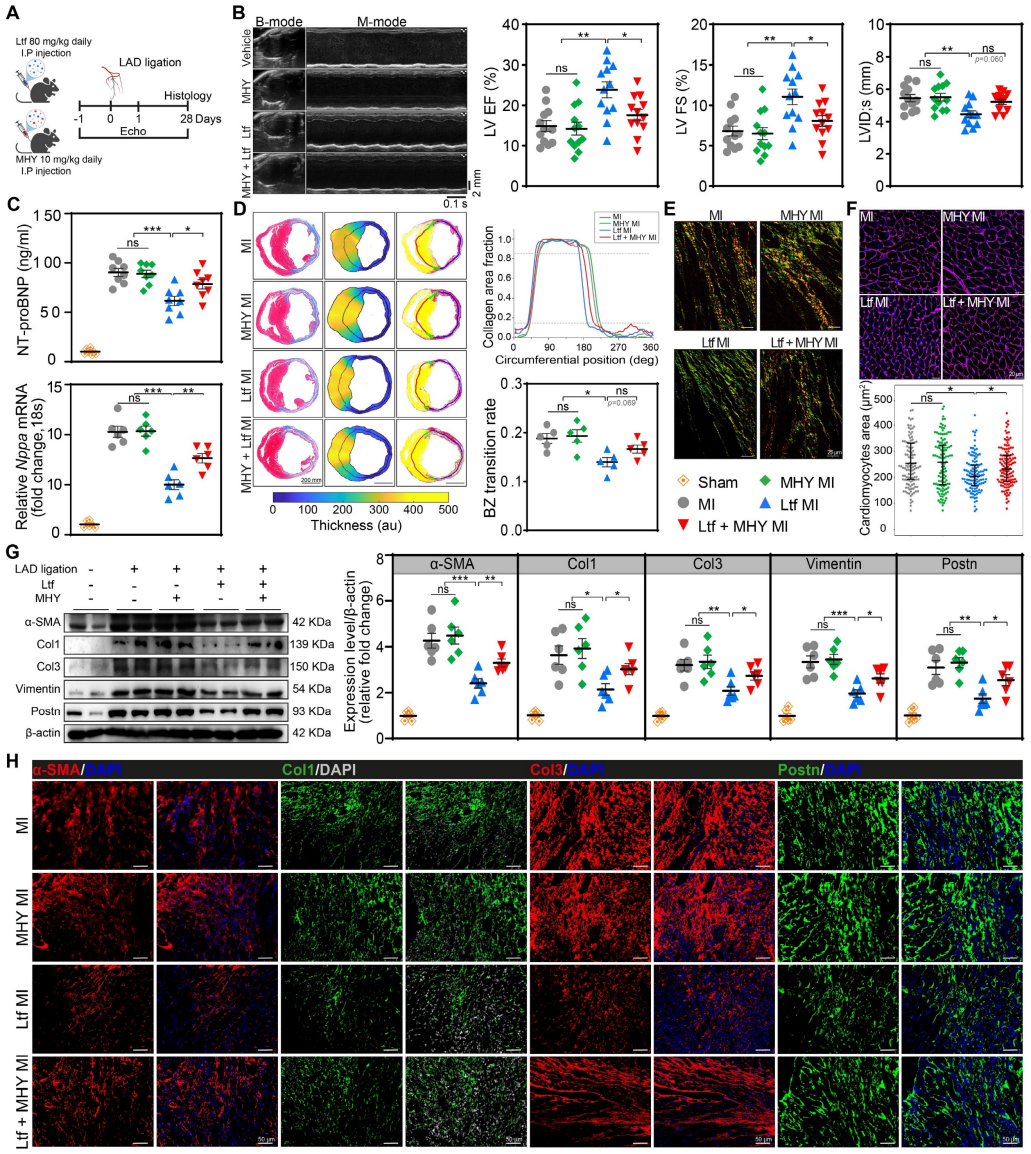
**mTORC1 agonist reverses cardiac protection of Ltf after MI *in vivo*. A**. Schematic of MI model in mice cotreated with Ltf and MHY1485. **B**. Representative echocardiographic images and quantificational related indicators 4 weeks after MI. n = 12/ea. **C.** The expression level of heart failure-associated indicators. NT-proBNP was tested in serum (up, n = 8/ea.), and *Nppa* in heart tissue (down, n = 6/ea.) 4 weeks post-MI. **D**. Representative images of Masson trichrome staining and picrosirius red-staining analyzed by pipeline. Relative quantifications are shown right. n = 5/ea. **E**. Representative of picrosirius red-staining under polarized microscopy in border zone. **F**. Representative WGA staining of cardiomyocytes in remote zone. Quantificational cardiomyocytes area is shown down to the graph. n = 5/ea. **G**. Western blot analysis and quantifications of α-SMA, Col1, Col3, Vimentin and Postn in corresponding subgroups. n = 6/ea. **H**. Representative immunofluorescent micrographs of indicated fibrosis-related proteins in injured myocardium at day 7 post-MI. Data are presented as mean ± SEM. **B**, **C, D** and **G**, by one-way ANOVA followed by Bonferroni post hoc test; **F**, by Mann-Whitney non-parametric test, *P < 0.05, **P < 0.001, ***P < 0.001 compared with corresponding group.

For further verification of whether Ltf attenuated adverse cardiac remodeling post-MI via mTORC1/S6K/eIF-4B axis, MHY1485 was administered intraperitoneally with a dosage of 10 mg/kg daily in mice (**Figure 7A**). Results showed that MHY1485 was not cardiotoxic after intraperitoneal administration over the study period with regard to no alteration in cardiac function, fibrosis and cardiomyocytes size among all sham groups (**Figure S13**). The suppressed mTORC1/S6K/eIF-4B signaling pathway by Ltf was significantly reversed after cotreatment with MHY1485 post-MI (**Figure S14**). Coadministration of Ltf and MHY1485 showed a downtrend of survival rate in MI mice compared to Ltf-treated MI group (**Figure S15A**). Functionally, cotreatment with MHY1485 abolished the optimal cardiac function mediated by Ltf in terms of ejection fraction, fractional shortening and ventricular size after MI (**Figure 7B**; **Figure S15B-E** and **Table S3**). Moreover, the beneficial effect of Ltf on attenuating the incidence of heart failure was counteracted by cotreatment with MHY1485, which presented with higher level of NT-proBNP, *Nppa*, *Nppb*, heart weight/tibia length and lung wet/dry ratio compared with Ltf-administrated MI group (**Figure 7C**; **Figure S15F-H**). These results verify that administration of Ltf improve cardiac function mainly through mTORC1/S6K/eIF-4B pathway.

Concerning the morphological features, Masson trichrome-staining and picrosirius red staining revealed that smaller border zone expansion and thinner scar thickness in Ltf-treated mice were partially reversed after co-administration with MHY1485 (**Figure 7D**; **Figure S16A**). Infarct size and fibrosis area in remote zone were similar among groups (**Figure S16A-B**). Moreover, collagen composition in border area was primary dominated by immature collagen under treatment with Ltf, while this composition was disrupted by intervention with MHY1485, as indicated by abundant mature collagen (**Figure 7E**; **Figure S16C**). Subsequently, Ltf-mediated alleviation of cardiomyocytes hypertrophy post-MI was abolished after cotreatment with MHY1485, owing to more severe fibrosis (**Figure 7F**). These data suggest that the effect of Ltf on suppressing adverse cardiac remodeling through inhibiting cardiac fibrosis was majorly mediated by mTORC1/S6K/eIF-4B signaling pathway.

Additionally, administration of MHY1485 counteracted the suppression of fibrosis-related genes (*α-SMA*, *Col1A1*, *Col3A1, Postn* and *Fn1*) induced by Ltf in MI mice (**Figure S17A**). Furthermore, the downregulation of α-SMA, Col1, Col3, Postn and Vimentin mediated by Ltf was partly reversed after co-administration of MHY1485 post-MI (**Figure 7G**). The immunofluorescence staining further confirmed that the effect of Ltf on repressing fibrosis was diminished after intervention with MHY1485 (**Figure 7H**; **Figure S17B**).

Taken together, our results indicate that Ltf attenuates fibrotic remodeling and improve outcomes after MI via inhibiting mTORC1/S6K/eIF-4B signaling pathway.

## Discussion

**Figure 8:**
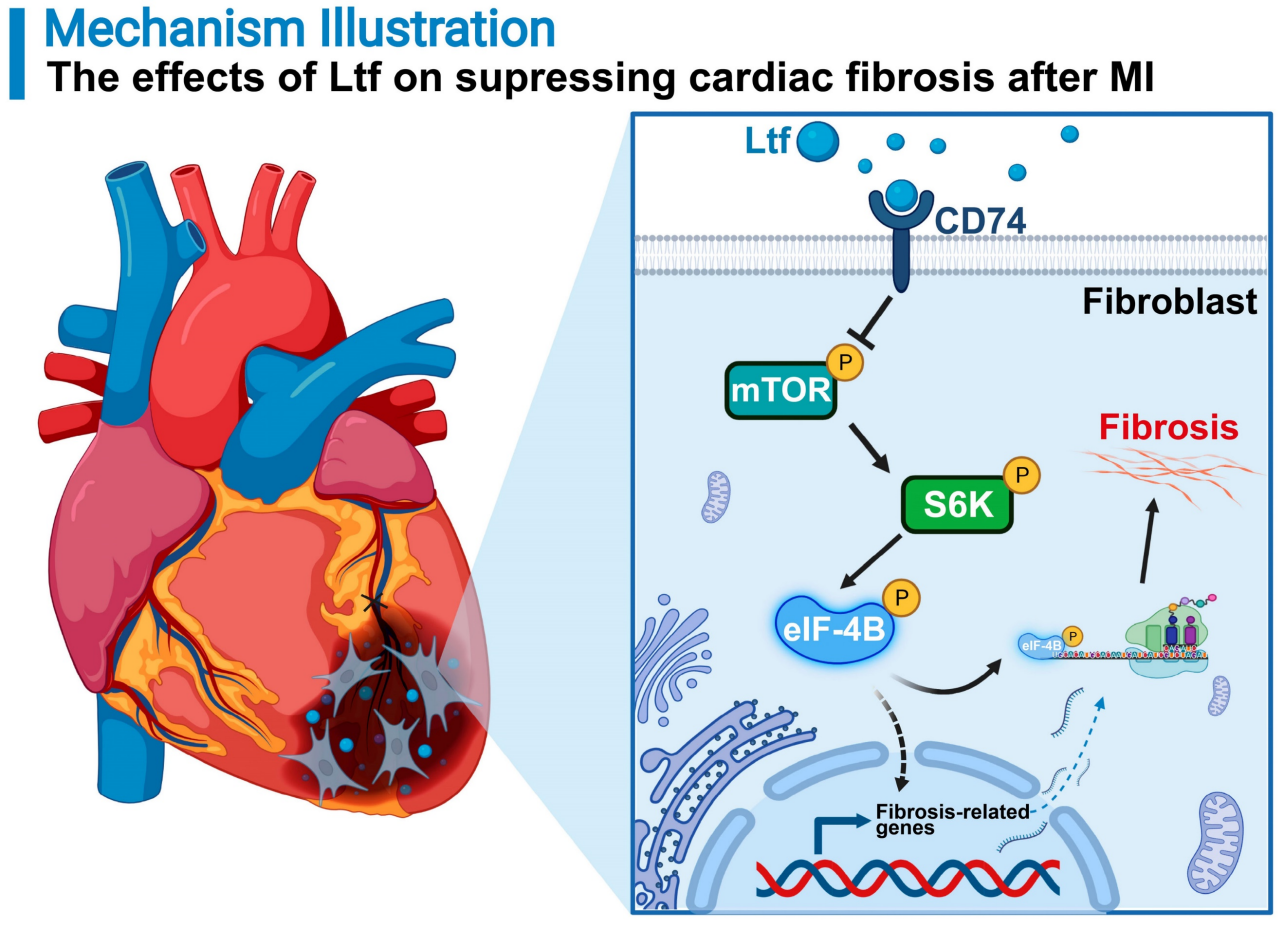
**A proposed model of how Ltf attenuates adverse cardiac remodeling via suppressing cardiac fibrosis.** MI stimuli triggers sustainable fibrotic process detrimental to cardiac functional recovery. Administration of Ltf alleviates adverse remodeling and improves cardiac function primarily via inhibiting excessive fibrosis after MI. Mechanistically, Ltf directly interacts with membrane receptor CD74 on fibroblasts, which subsequently represses the activation of mTORC1/S6K/eIF-4B, and in turn downregulates fibrosis-related genes, ultimately leading to suppression detrimental collagen-rich extracellular matrix deposition.

In this study, we show that Ltf can restrain cardiac fibrosis, alleviate cardiac remodeling, and improve cardiac function through suppressing mTORC1/S6K/eIF-4B signaling pathway post MI in mice (**Figure 8**). Previous studies have expanded the value of Ltf in pathophysiological functions referring to regulation of metabolism, inflammation, apoptosis process and oxidative stress in various disorders 15,17,18. To our knowledge, this is the first report to illustrate the protective effects of Ltf on MI injury. Mechanistically, Ltf can directly interact with CD74, which in turn represses mTORC1/S6K/eIF-4B axis to reduce the activation of fibroblasts. Our results hint that treatment with Ltf provides a novel promising strategy to improve outcomes of patients with MI.

Protective roles of Tregs have been verified in alleviating apoptotic process, local inflammation and cardiac fibrosis after MI, yet related mechanisms remain elusive 8,13,31. Based on label-free proteomic technology 32, we identified a number of remarkably altered proteins in cardiac Tregs after MI compared with spleen naïve T-cells. Functional analysis of these different expression proteins revealed that cardiac Tregs present their protective features by promoting cardiomyocytes survival, angiogenesis, wound healing, and attenuating cardiac fibrosis post -MI 12. Because Tregs can exert function via secreted factors 13, we focused on the proteins that were differentially expressed and proposed to be located in extracellular vesicles. Among them, Ltf was identified as an important factor. Ltf has been widely reported to exert positive effects on a spectrum of disorders related to inflammatory, oxidant and metabolism process 17. Circulating Ltf concentration differs in diseases, which is reduced in patients with obesity and diabetes 33-35. In our study, the Ltf level in both circulation and heart tissue were severely reduced after MI, while the reasons for the decrease of Ltf level in circulation and myocardium tissue require further research, probably due to the change of metabolism and immune response post-MI 17.

Prior study has demonstrated Ltf contributes protective effects on acute kidney injury and renal fibrosis 15. Our gain-function study of Ltf via exogenous supplementation suggested that Ltf could alleviate detrimental cardiac remodeling, reduce fibrosis, and improve cardiac function after MI injury. Excessive fibrosis exacerbates passive tissue stiffness and afterload elevation, contributing to detrimental remodeling post-MI 36. Treatment with Ltf effectively decreased cardiac fibrosis and incidence of heart failure, which were indicated by reduction of scar thickness and optimal cardiac function respectively. What's more, border zone, as a most vulnerable area of heart after MI, bears gradual elevation of wall stress and occurs scar expansion, which in turn damages uninjured cardiomyocytes in remote area 4,36. We found that administration of Ltf resulted in smaller border zone expansion. Additionally, our results demonstrated treatment with Ltf contributed to collagen content and composition alterations featured with less collagen-rich extracellular matrix deposition and more compliant collagen fibers in injured area. Myofibroblasts are the primary contributor to pathological fibrosis process after MI. TGF-β, which is presumed as a major factor of promoting fibroblast to myofibroblast conversion, is remarkably upregulated post-MI 23. As expected, our investigations found that administration of Ltf attenuated TGF-β-induced transformation of cardiac fibroblast to myofibroblast *in vitro*, with suppressing the capacities of proliferation, collagen formation and migration. Collectively, our study confirms that treatment with Ltf alleviates adverse cardiac remodeling and improves cardiac function through inhibiting cardiac fibrosis, which is presented with thin scar thickness, limited border zone expansion and less collagen deposition.

Mechanistically, phosphorylation is the most common posttranslational modifications in proteins to regulate biological function 37. We adopted a phosphoproteomic analysis to verify the molecular mechanism underlying the implications of Ltf on attenuating cardiac fibrosis. The mTORC1/S6K/eIF-4B axis was identified as the primary signaling pathway and highly activated in CTR MI group compared with Ltf MI group. The mTORC1 signaling pathway has been widely reported as an essential role in activating fibroblasts to instigate fibrosis process in diverse disorders 28,38-40. Targeting mTORC1/S6K/eIF-4B signaling pathway can alleviate cardiac remodeling and heart failure in response to pressure overload conditions and myocardial infarction 2,41. We found that treatment with Ltf suppressed the activation of mTORC1/S6K/eIF-4B pathway induced by MI stimuli *in vivo* and TGF-β *in vitro*. Specific activation of mTORC1/S6K/eIF-4B pathway by MHY1485 reversed the inhibitory effects of Ltf on fibroblast to myofibroblast conversion induced by TGF-β. Simultaneously, co-administration of MHY1485 partially abolished the protective effects of Ltf after MI, achieved by thicker scar wall, larger border zone expansion, more collagen-rich extracellular matrix deposition and more severe cardiac dysfunction. These data prove that Ltf suppresses the activation of myofibroblasts to reduce detrimental remodeling after MI primarily through suppressing mTORC1/S6K/eIF-4B pathway.

To our knowledge, few studies have comprehensively illustrated the impact of Ltf on fibrosis. To confirm the upstream of mTORC1/S6K/eIF-4B pathway which Ltf may interact with, an upstream-analysis via IPA was performed. As a result, CD74 was predicted as an upstream receptor for Ltf. CD74 is a membrane protein initially thought to be a chaperone of major histocompatibility complex class II, and has been verified the function on regulating proliferation and inflammation, promoting survival and wound healing 42-44. Recent work has demonstrated CD74 is critical for mediating antifibrosis process in various diseases 45,46. Moreover, CD74 has been illustrated to stimulate the activation of cardioprotective AMPK pathway which could directly inhibit mTORC axis post ischemic cardiac injury 47,48. All these prior studies hint that CD74 may regulate mTORC pathway to mediate antifibrosis response. In the present study, we identified Ltf could interact with CD74, further resulting in the suppression of mTORC1/S6K/eIF-4B pathway to alleviate fibrosis process after MI. Alternatively, we found treatment with Ltf increased the expression of CD74, which in turn enhanced the cardiac protective effects of Ltf.

Our study also bears significant clinical implications. Cardiac fibrosis is an irreversible pathological response to MI, and exaggerates the incidence of heart failure. Our work demonstrated the circulating Ltf level was decreased during MI process, and that administration of Ltf significantly alleviated adverse cardiac remodeling to improve cardiac function. Clinical interventions with Ltf have shown a favorable safety profile and potential health implications in various diseases 17. Furthermore, other research has identified Ltf as an effective therapy to inhibit apoptosis after injury 15,16. Combining the beneficial implications of Ltf described previously, we believe administration of Ltf can improve the outcomes of patients with MI.

There exist several limitations in the current study. First, the reasons for the decline of circulating and tissue Ltf level post-MI remains largely unknown, which needs further investigation. Second, although utilization of exogenous Ltf supplementation as gain-of function enhances the clinical translational value, mice models with Tregs specific overexpression of Ltf are more convincing to illustrate the function of Ltf. Third, the present study lacks reverse verification experiments on loss of function via *Ltf* gene knockout mice. However, we provided clear evidence illustrating that Ltf exerted remarkably cardiac protective function via suppressing detrimental remodeling after MI, an effect of great clinical significance.

In summary, our work reveals a novel effect of Ltf on suppressing adverse cardiac remodeling upon MI in that Ltf orchestrates the cardiac fibrosis process via suppressing proliferation, pro-fibrosis and migration capacities of myofibroblasts. Mechanistically, Ltf interacts with CD74, which in turn represses the activation of mTORC1/S6K/eIF-4B axis to depress cardiac fibrosis after MI. More broadly, our study provides great translational implication for the treatment of patients with MI.

## Materials and Methods

### Materials availability

A detailed description of materials and methods are given in the Supplemental Materials and are accessible from the corresponding author upon reasonable request.

### Statistical analysis

All quantitative data were shown with mean ± SEM. The normality of data was examined by Shapiro-Wilk normality test. Data with normally distribution was analyzed with unpaired Student *t* test or ANOVA followed by Bonferroni post hoc analysis for comparisons between 2 groups or multi-groups (>2 groups) respectively, while Mann-Whitney non-parametric tests was used for data featured non-normal distribution. Survival analysis were performed by the Kaplan-Meier methods and compared using log-rank (Mantel-Cox) test. Chi-square test was adopted for Categorical data. A *p*< 0.05 was regarded as statistical significance. Statistical analysis was performed via GraphPad Prism 9.0 (GraphPad Prism Software, Inc, San Diego, CA).

## Supplementary Material

Supplementary materials and methods, figures and tables.

## Abbreviations

MI: myocardial infarction
CHD: coronary heart disease
HF: heart failure
Ltf: lactoferrin
LAD: left anterior descending
CTR: control
Tregs: regulatory T-cells
TGF-β: transforming growth factor-β
TF: transcript factor
PCA: principal component analysis
ES: enrichment score
FDR: false-discovery rate
DEP: differentially expressed protein
GSEA: gene set enrichment analysis
GO: gene ontology
WGA: wheat germ agglutinin
DMP: differently modified phosphoprotein
IPA: ingenuity pathway analysis
α-SMA: α-smooth muscle actin
Col1: collagen type 1
Col3: collagen type 3
MMP2: matrix metalloproteinase-2
MMP9: matrix metalloproteinase-9
Timp1: tissue inhibitor of metalloproteinase-1
LV: left ventricular
EF: ejection fraction
FS: fractional shortening
LVID: left ventricular internal diameter
au: arbitrary units
ns: no significance
ROI: region of interest
