# Supplementary material for: Lactoferrin attenuates cardiac fibrosis and cardiac remodeling after myocardial infarction via inhibiting mTORC1/S6K signaling pathway
Source: Theranostics. 2023 Jun 4;13(10):3419–33. doi: 10.7150/thno.85361 (PMC10283051; doi:10.7150/thno.85361)
Supplement: Supplementary file 1 — Supplementary materials and methods, figures and tables. [file thnov13p3419s1.pdf]

SUPPLEMENTAL MATERIAL

Lactoferrin attenuates cardiac fibrosis and cardiac remodeling after myocardial infarction via inhibiting mTORC1/S6K signaling pathway

Tianbao Ye[1,2#], Zhiwen Yan[3#], Cheng Chen[4#], Di Wang[1], Aiting Wang[2], Taixi Li[1], Boshen Yang[1], Xianting Ding[2*], Chengxing Shen[1*]

Affiliations:

[1]Department of Cardiology, Shanghai Sixth People's Hospital Affiliated to Shanghai Jiao Tong University School of Medicine, Shanghai 200233, China

[2]Institute for Personalized Medicine, School of Biomedical Engineering, Shanghai Jiao Tong University, Shanghai 200030, China

[3]Youth Science and Technology Innovation Studio of Shanghai Jiao Tong University School of Medicine, Shanghai 200233, China

[4]School of Medicine, Tongji University, Shanghai 200092, China

[#]These authors contributed equally to this work.

*Corresponding authors.

Chengxing Shen: Email: shencx@sjtu.edu.cn (C.S.), Tel/Fax: 086-021-64369181;

Xianting Ding: Email: dingxianting@sjtu.edu.cn (X.D.), Tel/Fax: 86-21-62932274

Materials and Methods

Animal

Mice used in this study were C57BL/6 mice (6-8 weeks old, male) purchased from SLRC Laboratory Animal Co., Ltd. (Shanghai, China) and bred in a specific pathogen-free condition. Animal studies were strictly performed in compliance with Animal Care and Use Committee of Shanghai Jiao Tong University. Besides, all animal procedures were followed by the National Institutes of Health Guideline Guide for the Care and Use of Laboratory Animals. Euthanasia in mice was achieved by carbon dioxide inhalation followed by cervical dislocation. No statistical approach was performed to predetermined sample size.

Drug administration

Lactoferrin (Sigma) was dissolved in PBS. Mice were intraperitoneally injected with lactoferrin at a dosage of 80 mg/kg once daily from the start of MI operation to the end of study period. Mice were treated with mTORC1/S6K/eIF-4B signaling pathway agonist MHY1485 (MCE) intraperitoneally at a dosage of 10 mg/kg daily from the day of surgery to the end of observational period. For cell stimulation, MHY1485 was dissolved in sterilized H_2O in assistance with ultrasonic.

Mouse model of MI

The MI operation was performed by permanent left anterior descending (LAD) artery ligation. The detailed operations of MI and sham surgery were described previously [1]. In brief, mice were anesthetized with 2% isoflurane inhalation. A small incision was made between the third and fourth intercostal rib to expose internal chest. Gentle pressure was given to help the heart to pop out the chest. LAD artery was ligated with a 6-0 silk suture at a level of 2 mm away from its origin. Successful occlusion of LAD artery was defined by immediate blanching of the anterior wall. The heart was quickly placed back into the thoracic cavity. Mice surviving less than 24 hours after MI procedure were excluded from the study. Sham operated mice were made according to the procedure without LAD artery ligation. All surgeries were carried out in a blinder manner.

Transthoracic echocardiographic analysis

Echocardiography was detected by Vevo 2100 instrument (Visualsonic) equipped with MS-400 probe to assess cardiac function in vivo. Hearts were visualized in M-mode from parasternal long-axis view or parasternal short -axis view at the level of two papillary muscles. Measurement was calculated by averaging of three consecutive cardiac cycles. Left ventricular internal diameters at the end of diastolic (LVEDD) and systolic (LVESD) were obtained according to M-mode. Ejection fraction (EF), Fractional shortening (FS), Left ventricular internal volume at the end of diastolic (LVEDV) and systolic (LVESV) were calculated automatically by echocardiographic software. Echocardiography measurements were run in a blinded manner.

Histology

Hearts were collected and fixed with 4% paraformaldehyde after perfused sufficiently with PBS. Hearts were dehydrated and embedded with paraffin. Then, hearts were cut into five consecutive sections (7 µm), 500 µm apart, starting from the apex to base of heart. For Masson's trichrome staining, consecutive five sections were performed per manufacturer's guideline (Sigma). Infarct size was determined by total infarct circumference/total left ventricular circumference. Meanwhile, the scar thickness was calculated using ImageJ software (1.52 V).

For picrosirius red staining, slides were performed with 0.1% sirius red (Sigma) at room temperature for 1 hour. Picrosirius red staining were viewed under polarized light microscopy to study collagen fiber distribution. Moreover, images were analyzed with custom-built semiautomated image processing pipeline that was established based on ventricular thickness and color segmentation with HSL color differentiation as previously described [2-4]. Briefly, the cross section of the heart is detected with inner (ventricular lumen) and outer (myocardial wall) boundaries. Ventricular wall thickness was calculated using a Eulerian solution to a pair of linear partial differential equations over the histological domain. The heart was divided into 120 circumferential partitions according to the inner and outer boundaries. HLS color space were used to identified myocardium (yellow: H30° -90°, S = 0.1-1.0, L = 0.1-0.93) and collagen (red: H330°-30°, S = 0.1-1.0, L = 0.1-0.93). The fraction of myocardium and fibrosis in area fractions and local wall thickness values were calculated using the image process. The fraction of collagen area per circumferential degree were calculated in the transition region. Border zone was characterized with the red/yellow ratio ranging from 0.15 to 0.85; infarct zone is defined as red/yellow ratio more than 0.85; while the remote zone is identified as red/yellow ratio less than 0.15. Eventually, the ratio of infarct length to total circumferential length (in degrees) was used to estimate the percent circumference of the infarct region.

Immunofluorescence staining

Freshly collected hearts were embedded in optimum cutting temperature (OCT) compound and cut into thin sections (7µm). The cryosections were blocked with 10% goat serum albumin with 0.03% TritonX-100 for 1 hour at room temperature after fixation/permeabilization. Then, slides were incubated with indicated primary antibodies overnight at 4 ℃. Following washing with PBS, the samples were stained with corresponding fluorescence secondary antibody for 2 hours at room temperature. Finally, nuclear was stained with DAPI (Invitrogen) for 10 minutes. Slides were viewed using fluorescent microscopy and fluorescent intensity was analyzed using ImageJ software (1.52 V).

WGA staining and cardiomyocytes area analysis

Wheat germ agglutinin (WGA) staining was performed as previously described [5]. In brief, the cryosections were stained in 50 µg/mL WGA conjugated with Alexa Flour 488 (Invitrogen) at room temperature for 1 hour. For quantifications of short-axis cardiomyocytes size, four to six independent hearts in each group were capture at 20X magnification in injured myocardium area. Each image was divided into 20 fields of view equally, and the average cell size in each field was calculated by using ImageJ software

(1.52 V). A minimum of 500 cells per sample were calculated.

Fibroblasts isolation

Adult hearts were dissected freshly, minced into small pieces. Subsequently, the pieces were digest with 0.1% type II collagenase (Worthington Biochemical) for 1 hour at 37 ℃ with shaking. After digestion, the supernatants were passed through 70 μm nylon mesh filter, and were plated for 2 hours for CFs adherent. Next, the medium was discarded and replaced with fresh culture medium.

Flow cytometry and FACS sorting

The isolation of heart-infiltrating immune cells was performed as previously described [6]. Briefly, the fresh heart tissue was collected and minced into small pieces, then subsequently digested with 0.1% type II collagenase (Worthington Biochemical) in HEPES buffer for 1 hour at 37 ℃ with shaking. Cells suspensions were passed through 70 μm cell strainers and mononuclear cells were purified by Ficoll (Merck). For spleen single cell suspension, fresh spleen was ground in cold-PBS buffer and filtered with 70 μm cell strainers. The purified mononuclear cells were stained with CD45-APC-R700 (BD Pharmingen), CD3-BV421 (BD Pharmingen), CD4-BV510 (BD Pharmingen). Intracellular staining of Foxp3-Alexa Flour 647 (BD Pharmingen) was performed using Intracellular Fixation & Permeabilization buffer (Invitrogen). Flow cytometry was performed using BD FACSA instrument and analyzed via Flowjo software.

Cell culture, stimulation and immunostaining

Cells were cultured at 37 ℃ in an incubator containing 5% CO_2. CFs were cultured in DMEM-F12 supplemented with 10% fetal bovine serum (FBS) and 1% penicillin/streptomycin. The passage 2 (P2) generation of CFs were used for experiments. For stimulation, P2 CFs were serum starved for 24 hours and then treated with 10 ng/ml TGF-β (PepProTech), 100 μg/mL Ltf (Sigma), or 2 μM MHY1485 (MCE) for appropriate time.

After 24 hours treatments, CFs were fixed with 4% paraformaldehyde and permeabilized with 0.03 % TritonX-100 for 20 minutes, and next blocked with 5% BSA with 1% donkey serum for 30 minutes. CFs were subsequently stained with indicated primary antibodies at 4 ℃ overnight. Afterwards, CFs were incubated with labeling fluorescent secondary antibody for 1 hour at room temperature and nuclear was stained with DAPI (Invitrogen). Fluorescent images were obtained by fluorescent microscopy and fluorescent intensity of cells was quantified using ImageJ software (1.52 V).

Proteomics and analysis

The label-free proteomics was performed as previously described [7]. Five thousand Tregs sorted via FACS sorting were added into DDM lysis buffer (0.1% DDM, 1 mM TECP and 2 mM CAA) in low protein-binding tubes and ultrasonicated for 1 hour followed by heated 1 hour at 60 ℃. Then the extracted proteins were digested with trypsin (ThermoFisher) at a ratio of 1:10 at 37 ℃ overnight, and the reaction was stopped by adding formic acid (FA) to a final concentration of 1%. The extracted peptides were dried

immediately and dissolved with 5 µl of 0.1% FA before performing LC/MS/MS analysis.

For phosphoproteomics, samples were prepared according to Filter-Aided Sample Preparation (FASP). Briefly, tissues were lysed with SDT buffer (2% SDS, 0.1 M DTT in 100 mM Tris/HCl, pH 7.6) supplemented with complete protease inhibitor (Roche) and phosphatase inhibitor (Roche) and heated at 95 °C for 5 minutes. Equal amount of protein lysates were added into Ultracel-30 tube (Millipore) to replace SDT buffer with UA buffer (8 M Urea in 0.1 M Tris/HCl, pH 8.5), and incubated with Iodacetamide (IAA) solution (50 mM in 50 mM NH4HCO3 solution) for 30 minutes in dark at room temperature. After washing with 50 mM NH4HCO3 solution, the extracted proteins were digested with trypsin at 37 °C overnight and terminated by adding FA to a final concentration of 1%. Phosphopeptides were enriched by high-selective Fe-NTA phosphopeptides enrichment kit (ThermoFisher) per manufacturer's instruction.

For data-independence-analysis (DIA), mixture of peptides derived from each sample was loaded into High-pH, reversed-phase fractionation spin column (ThermoFisher) per manufacturer's instruction to obtained fractions for building spectra library for DIA analysis. peptides were quantified equally using pierce quantitative fluorometric peptide assay (ThermoFisher) according to manufacturer's guideline prior to LC/MS measurement. Peptides were identified with Q-Exactive™ Plus Hybrid Quadrupole-Orbitrap™ (ThermoFisher).

The MS raw files were processed with Skyline searched against the mice UniProt database [8,9]. The different expression proteins or different modification proteins (|fold change| > 1.5, p<0.05) were analyzed with Gene ontology (GO) terms using DAVID (https://david.ncifcrf.gov/), and KEGG mapper (https://www.kegg.jp/). Gene Set Enrichment Analysis was performed using GSEA (http://www.gsea-msigdb.org/). Kinase substrate enrichment analysis was performed using Cytoscape (https://cytoscape.org/).

RT-PCR

Total RNA from tissues or cells was extracted using Total RNA Isolation Kit (Vazyme), per manufacturer's guidance. cDNA was prepared using HiScript III 1st Strand cDNA Synthesis Kit (Vazyme), per manufacturer's protocol. Quantification reverse transcription polymerase chain reaction (RT-PCR) was conducted using Universal SYBR Green Mater Mix (Vazyme). The primers for RT-PCR were presented (**Table S4**). 18s served as a housekeeping gene. Standard comparative CT method was used for calculation of gene expression.

Western blotting

Total proteins were extracted from cells or heart tissues using RIPA buffer supplementary with complete protease inhibitor (Roche) and phosphatase inhibitor (Roche, Germany). Protein concentration was quantified using Pierce BCA Protein Assay Kit (ThermoFisher). Equal amount of protein was added to SDS/PAGE Gels. After separation, SDS/PAGE Gels proteins were transferred to Polyvinylidene difluoride (PVDF) membranes and blocked with 5% BSA/TBS. The membranes were incubated with indicated primary antibodies at 4 ℃ overnight (**Table S5**). Membranes were next incubated with horseradish peroxidase-conjugated secondary antibody for 2 hours. The membranes were explored

using Tanon instrument and calculated with ImageJ software (1.52 V).

Coimmunoprecipitation

P2 CFs were treated with Ltf for 10 minutes. Cells were washed with PBS gently and lysed with immunoprecipitation lysis buffer (2.5 mM PH7.4 Tris; 150 mM NaCl; 1 mM EDTA; 1% NP40; 5% glycerol; protease inhibitor cocktail). The co-immunoprecipitation was performed using Pierce Co-IP kit (ThermoFisher) according to manufacturer's guidance. Briefly, 10 μg antibody against Ltf or CD74 was transferred into the AminLink Plus Coupling Resin. Protein extracts were transferred into the provided column and incubated at 4 ℃ overnight. The eluted samples were analyzed via western blotting. Negative control was served by IgG to evaluate nonspecific binding. For cell free in tube assays, 3 μg mouse recombinant Ltf (MCE) and 3 μg mouse recombinant CD74 (MCE) were added in lysis buffer, and then the mixture was rocked at 4 ℃ overnight prior to co-immunoprecipitation.

TUNEL assay

Cryosections (7 μm thickness) were used to TdT-mediated dUTP Nick-End Labeling (TUNEL) assay. TUNEL staining was performed by TUNEL Kit (Roche) following manufacturer's instruction. Images were acquired by fluorescence microscopy.

CCK8 assay

P2 CFs were seeded into 96-well plates. Cell Counting Kit-8 (CCK-8) (DOJINDO) were used to analysis the cell population viability according to corresponding time points. The absorbance was calculated at 450 nm via the microplate reader (BioTek).

TTC assay

The infarct heart was transversely sectioned into five pieces from the apex to base with one piece locating at the ligation site 24 hours post-MI, and then incubated with 1.0% 2,3,5-triphenyltetrazolium chloride (TTC, Sigma-Aldrich) for 20 minutes at 37°C (protect from light). The sections were next fixed with 4% paraformaldehyde for 5 minutes. Subsequently, Viable myocardium presented red while infarcted area revealed white. The infarct size was analyzed with ImageJ software (1.52 V).

Migration assay

An 8-μm transwell system (Corning Costar) was used to conducted migratory assays. CFs were plated at 3×10^4 cells per well to the top chamber with serum-free medium. The bottom well was added with complete medium. Following 24 hours of incubation, cells that did not migrate and left inside the upper chamber were wiped off. The migratory CFs were fixed with 4% paraformaldehyde before being stained with 0.01% Crystal Violet (Sigma), and then counted using microscopy at six random fields.

ELISA assay

Ltf levels were examined by enzyme-linked immunosorbent assay kit (ELISA) (Abcam; CUSABIO). For tissue sample preparation, heart tissue was treated with ice-cold Lysis Buffer (10 mM PH = 8.0 Tris; 130 mM NaCl; 1% TritonX-100; protease inhibitor cocktail)

for 60 minutes, then centrifuged and collected supernatant. The measurements were conducted following manufacturer's introduction. Read the plate at 450 nm absorbance.

References

1. Gao E, Lei YH, Shang X, Huang ZM, Zuo L, Boucher M, et al. A novel and efficient model of coronary artery ligation and myocardial infarction in the mouse. Circ Res. 2010; 107: 1445-53.

2. Bersi MR, Khosravi R, Wujciak AJ, Harrison DG, Humphrey JD. Differential cell-matrix mechanoadaptations and inflammation drive regional propensities to aortic fibrosis, aneurysm or dissection in hypertension. J R Soc Interface. 2017; 14.

3. Schroer AK, Bersi MR, Clark CR, Zhang Q, Sanders LH, Hatzopoulos AK, et al. Cadherin-11 blockade reduces inflammation-driven fibrotic remodeling and improves outcomes after myocardial infarction. JCI Insight. 2019; 4.

4. Snider JC, Riley LA, Mallory NT, Bersi MR, Umbarkar P, Gautam R, et al. Targeting 5-HT2B Receptor Signaling Prevents Border Zone Expansion and Improves Microstructural Remodeling After Myocardial Infarction. Circulation. 2021; 143: 1317-30.

5. Nakada Y, Canseco DC, Thet S, Abdisalaam S, Asaithamby A, Santos CX, et al. Hypoxia induces heart regeneration in adult mice. Nature. 2017; 541: 222-7.

6. Xia N, Jiao J, Tang TT, Lv BJ, Lu YZ, Wang KJ, et al. Activated regulatory T-cells attenuate myocardial ischaemia/reperfusion injury through a CD39-dependent mechanism. Clin Sci (Lond). 2015; 128: 679-93.

7. Wang L, Abdulla A, Wang A, Warden AR, Ahmad KZ, Xin Y, et al. Sickle-like Inertial Microfluidic System for Online Rare Cell Separation and Tandem Label-Free Quantitative Proteomics (Orcs-Proteomics). Anal Chem. 2022; 94: 6026-35.

8. Lin CC, Kitagawa M, Tang X, Hou MH, Wu J, Qu DC, et al. CoA synthase regulates mitotic fidelity via CBP-mediated acetylation. Nat Commun. 2018; 9: 1039.

9. Wood AJ, Lin CH, Li M, Nishtala K, Alaei S, Rossello F, et al. FKRP-dependent glycosylation of fibronectin regulates muscle pathology in muscular dystrophy. Nat Commun. 2021; 12: 2951.

Supplemental Figures

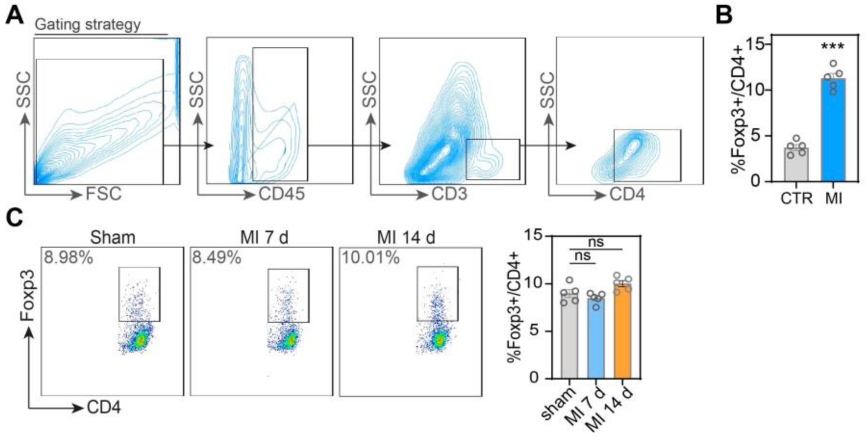

Figure S1. Tregs population in heart is increased at day 7 post-MI. **A.** Gating strategy for Tregs. **B**. Quantification of Tregs population in heart at day 7 post-MI. n = 5/ea. **C.** Flowcytometry analysis of Tregs population in spleen at different time points. Quantification is shown right. n = 5/ea. Data are presented as mean ± SEM. **A**, by unpaired student *t* test; **C**, by one-way ANOVA followed by Bonferroni post hoc test, ***P < 0.001 compared with control group. ns, no significance.

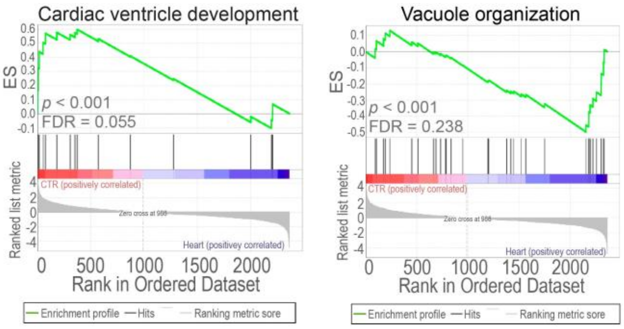

Figure S2. GSEA analysis via GO biological process database. Adjusted p-values and FDR are added to each enrichment dataset.

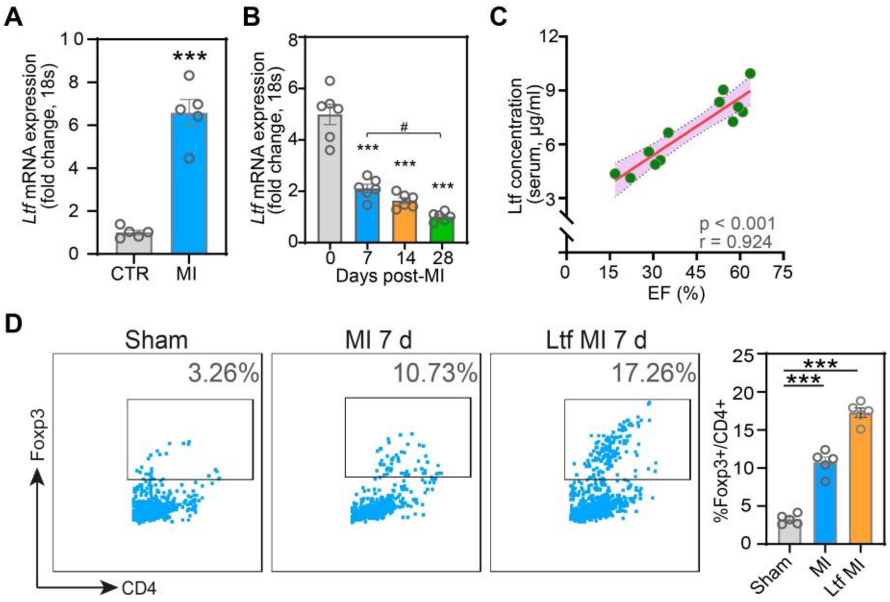

Figure S3. Ltf is downregulated in heart after MI, with compensatory increase in Tregs. A and B. mRNA expression of *Ltf* in naïve Tregs and cardiac Tregs at day 7 post-MI (**A**, n = 5/ea), and in border zone at different time points after MI (**B**, n = 6/ea). **C**. Correlation between circulating Ltf concentration and EF in mice with MI. p-value and correlation coefficient are presented. **D**. Flowcytometry assays of cardiac Tregs population in corresponding conditions, with quantification right. n = 5/ea. Data are presented as mean ± SEM. **A**, by unpaired student *t* test; **B** and **D**, by one-way ANOVA followed by Bonferroni post hoc test; **C**, Pearson correlation test, *P < 0.05, ***P < 0.001 compared with control group; $^{\#}$P < 0.05, compared with indicated group. Ltf MI, mice treated with Ltf under MI operation.

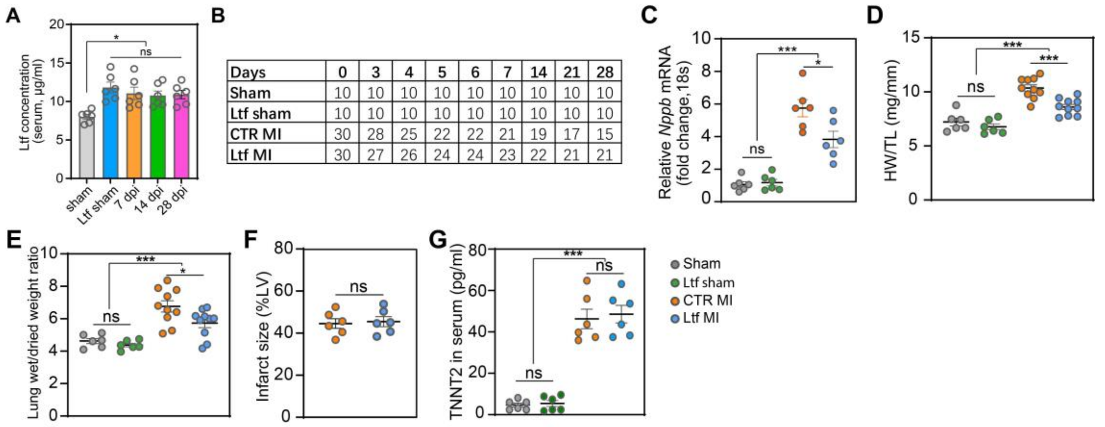

Days	0	3	4	5	6	7	14	21	28
Sham	10	10	10	10	10	10	10	10	10
Ltf sham	10	10	10	10	10	10	10	10	10
CTR MI	30	28	25	22	22	21	19	17	15
Ltf MI	30	27	26	24	24	23	22	21	21

Figure S4. Ltf attenuates MI-induced heart failure without effect on infarct size. **A**. The Ltf protein level in serum following intraperitoneal administration of Ltf at different time points. n = 5-6/ea. **B**. Overall survival number in the subgroups at different time points within 28 days respectively. **C**. Relative mRNA expression of *Nppb* in mouse hearts. **D**. Heart weight–to–tibia length ratio (HW/TL). n = 6-10/ea. **E**. Wet lung weight–to–dried lung weight ratio. n = 6-10/ea. **F**. Quantification of infarct size according to TTC assay. n = 6/ea. **G**. Circulating TNNT2 level at day 1 post-MI. n = 6/ea. Data are presented as mean ± SEM. **F**, by unpaired student t test; **A**, **C**, **D**, **E** and **G**, by one-way ANOVA followed by Bonferroni post hoc test, *P < 0.05, ***P < 0.001 compared with control group. dpi, day post injury.

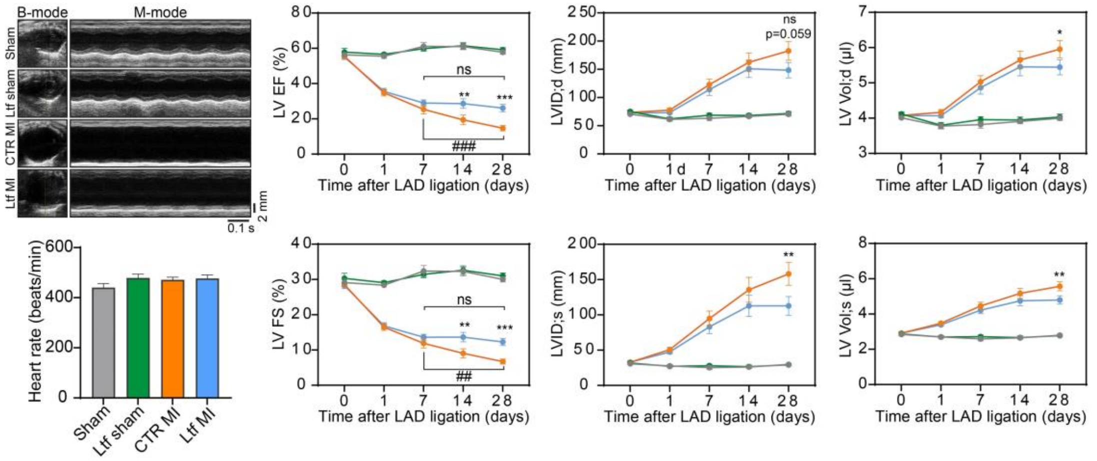

Figure S5. Serial echocardiographic were acquired using short-axis mode at day 0, 1, 7, 14, and 28 after MI with relative quantifications next (n = 10-15/ea). Data are presented as mean ± SEM, by two-way ANOVA followed by Bonferroni post hoc test, *P < 0.05, **P < 0.01, ***P < 0.001 compared with control MI group; $^{\#\#}$P < 0.01, $^{\#\#\#}$P < 0.001 compared with day 7 in CTR MI group.

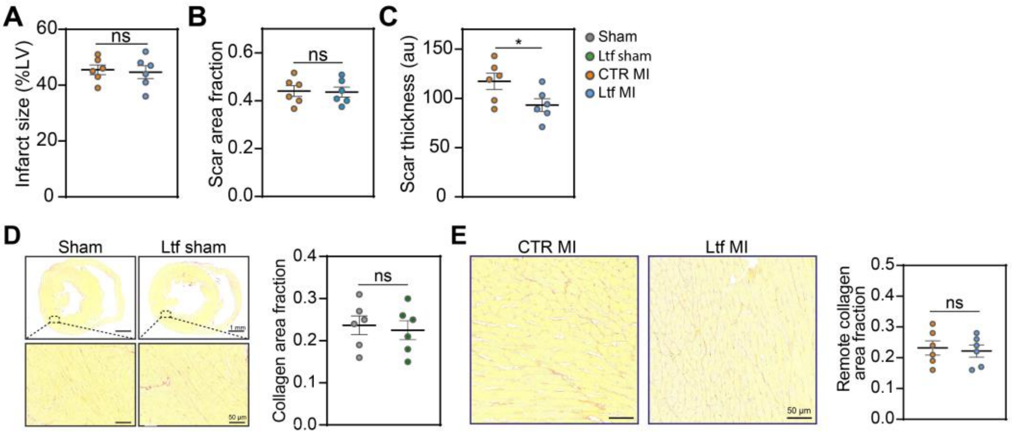

Figure S6. **A**. Quantification of infarct size according to Masson trichrome-staining. n = 6/ea. **B** and **C**. Statistic calculations of infarct size (**B**, n = 6/ea.) and scar thickness (**C**, n = 6/ea.) referred to picrosirius red staining at day 28 after MI. **D**. Representative images (left) and quantification (right) of picrosirius red staining between sham groups. n = 6/ea. **E**. Representative images (left) and quantification (right) of picrosirius red staining in remote uninjured area 28 days post-MI. n = 6/ea. Data are presented as mean ± SEM, by unpaired student t test, *P < 0.05 compared with control group.

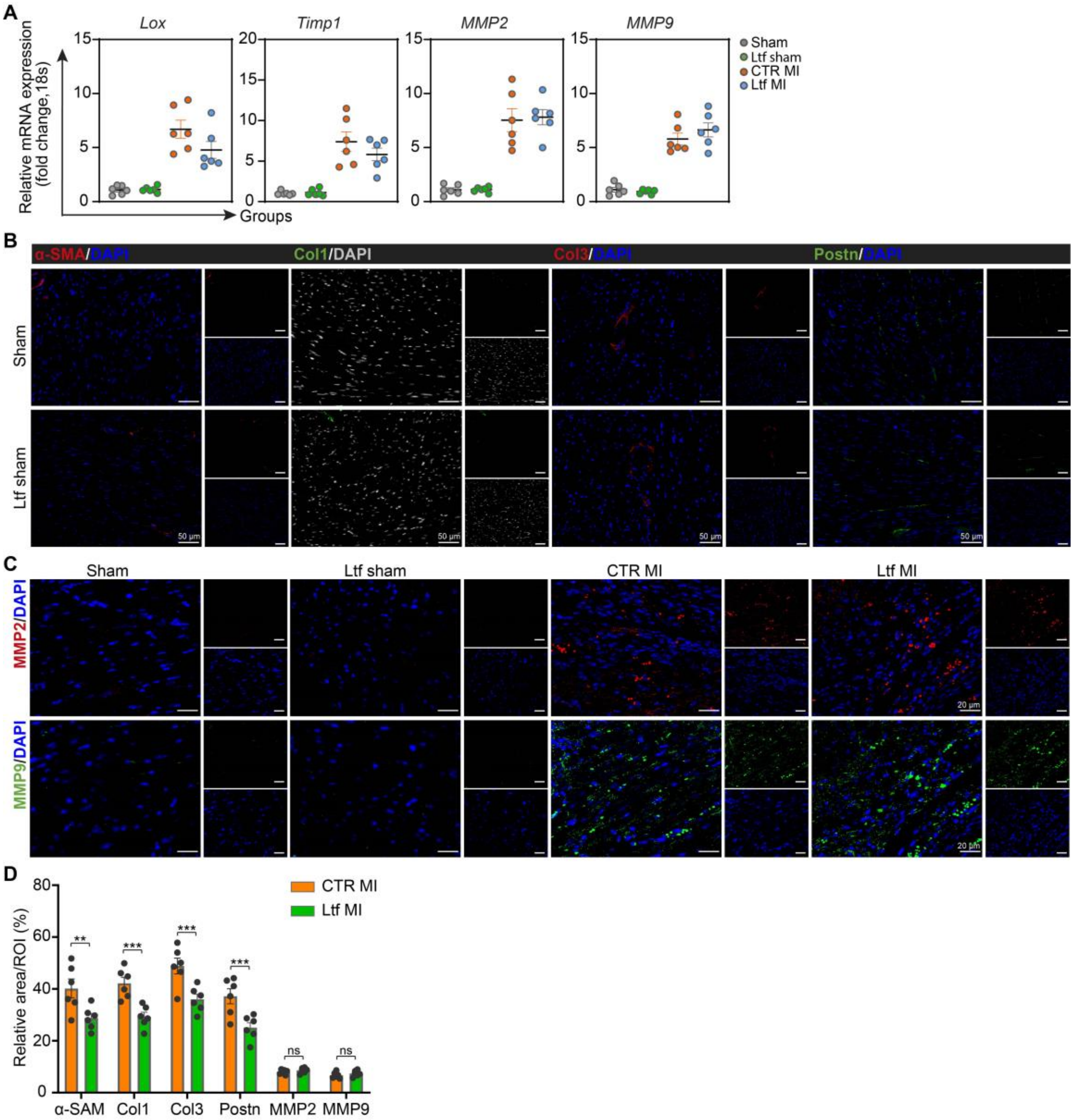

Figure S7. **A.** mRNA expression of fibrosis-associated enzymes genes**.** n = 6/ea. **B.** Immunofluorescence staining of α-SMA, Col1, Col3 and Postn in sham groups. **C.** Representative images of immunofluorescence staining of MMP2 and MMP9 in injury myocardium. **D**. Relative quantification of immunofluorescence staining. n = 5-6/ea. Data are presented as mean ± SEM. **A** (*LOX*, *MMP2*, *MMP9*) by one-way ANOVA followed by Bonferroni post hoc test; **A** (*Timp1*), by Mann–Whitney non-parametric test; **D**, by unpaired student t test, ***P < 0.001 compared with corresponding group.

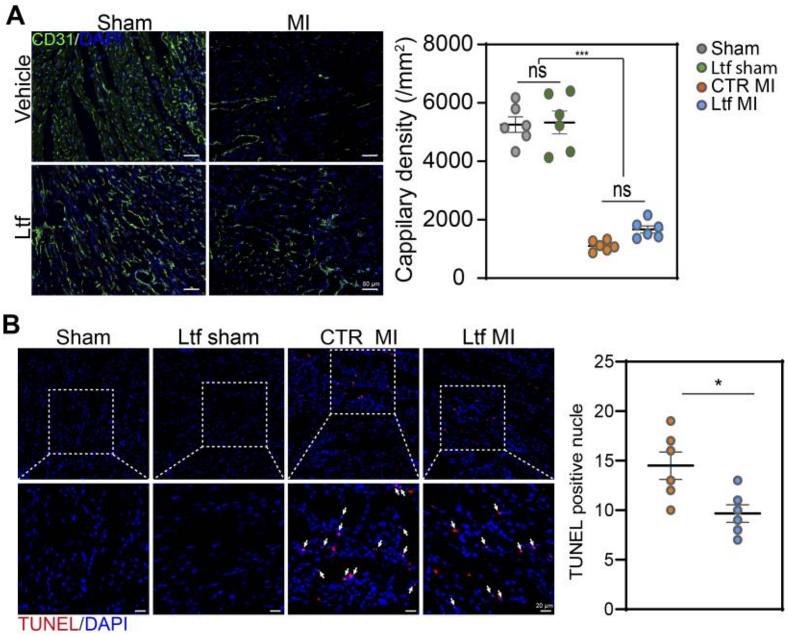

Figure S8. **Ltf improves angiogenesis and cardiomyocytes survival after MI. A.** Representative images of immunofluorescence staining of microvascular with CD31 in injury myocardium. n = 6/ea. **B.** Representative micrograph of immunofluorescence staining of apoptotic cells with TUNEL. Quantification is shown next to graphs. n = 6/ea. Data are presented as mean ± SEM. **A**, by one-way ANOVA followed by Bonferroni post hoc test; **B**, by unpaired student t test, $*P < 0.05$, $***P < 0.001$ compared with corresponding group.

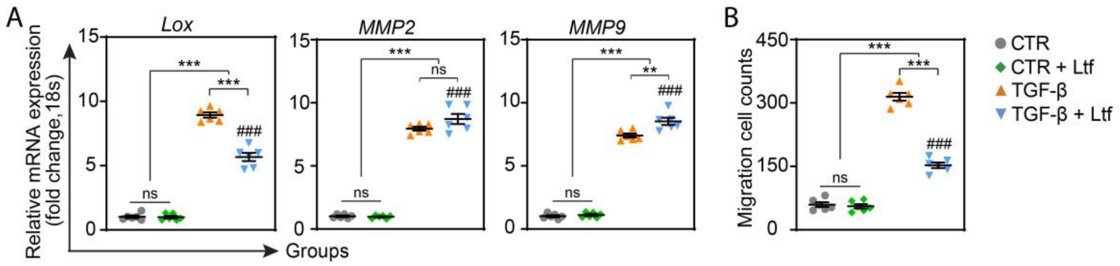

Figure S9. **Ltf partially inverts TGF-β-mediated transformation of fibroblast to myofibroblast. A.** Relative mRNA expression of indicated genes. n = 6/ea. **B.** Quantification of migratory cell number. n = 6/ea. Data are presented as mean ± SEM, by one-way ANOVA followed by Bonferroni post hoc test, *P < 0.05, **P < 0. 01, ***P < 0.001 compared with corresponding group. ###P < 0.001 compared CTR group.

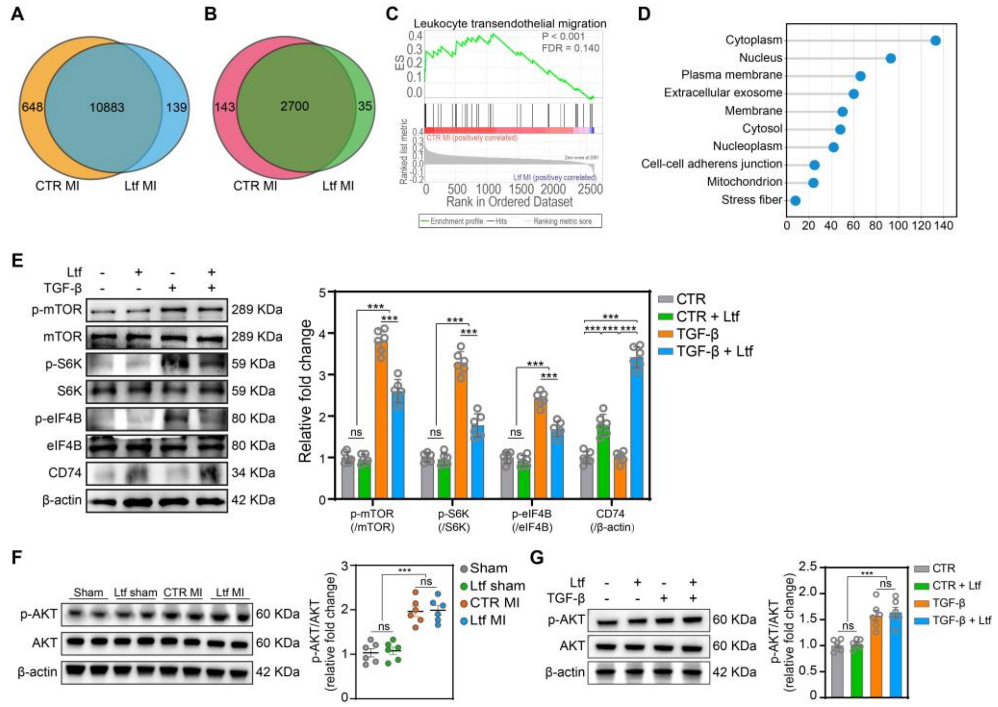

Figure S10. **A and B.** Venn diagram demonstrating the number of the phosphosites (**A**) and corresponding proteins (**B**). **C.** GSEA analysis via KEGG database. **D**. Cell component referred to GO terms of DMPs. **E**. Representative Western blot of indicated proteins. Quantification is shown next to graph. n = 6/ea. **F-G**. Western blot analysis of mTORC2 pathway assessed by phosphorylation AKT (S473) in mouse hearts (**F**) and fibroblasts (**G**) under different disposes. n = 6/ea. Data are presented as mean ± SEM, by one-way ANOVA followed by Bonferroni post hoc test, ***P < 0.001 compared with corresponding group.

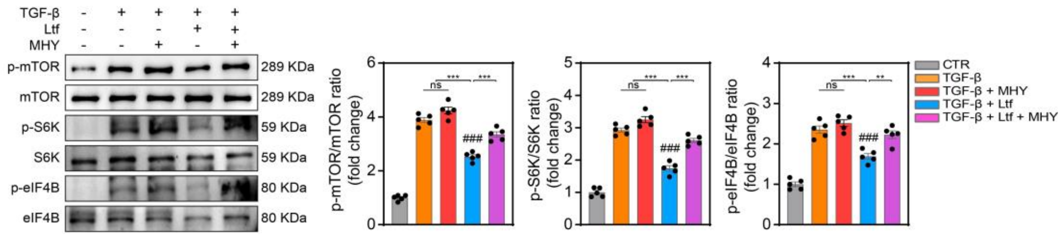

Figure S11. **MHY1485 restores the downregulation of mTORC1/S6K/eIF-4B pathway mediated by Ltf.** Representative of Western blot and quantificational blot images of indicated proteins in fibroblasts. n = 5-6/ea. Data are presented as mean ± SEM, by one-way ANOVA followed by Bonferroni post hoc test, ***P < 0.001 compared with corresponding group; $^{###}$P < 0.001 compared with control group.

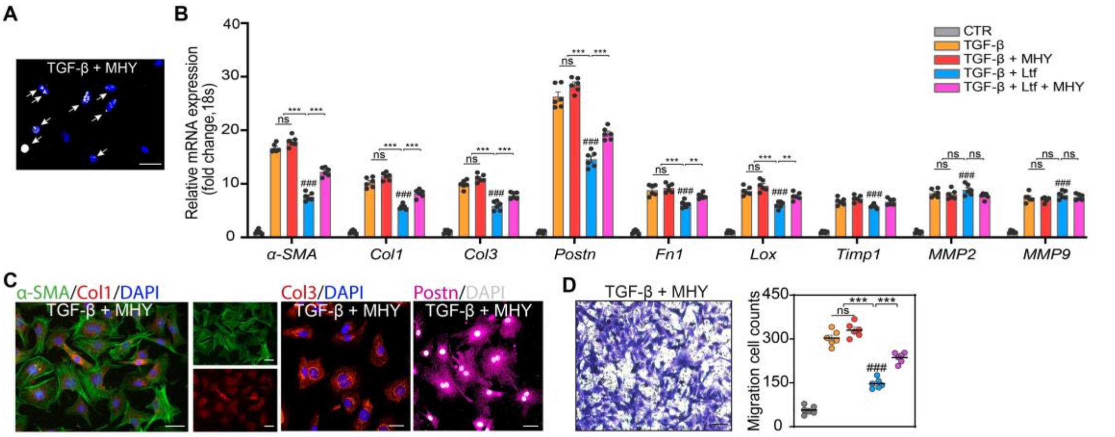

Figure S12. **A** Representative micrograph of immunofluorescence staining with Ki67. **B**. Relative mRNA expression of indicated genes. n = 6/ea. **C**. Representative immunofluorescent images of related proteins in cotreated with TGF-β and MHY1485 group. **D**. Representative image of transwell assay and relative quantification. n = 6/ea. Data are presented as mean ± SEM, by one-way ANOVA followed by Bonferroni post hoc test, ***P < 0.001 compared with corresponding group; $^{\#\#\#}$P < 0.001 compared with control group.

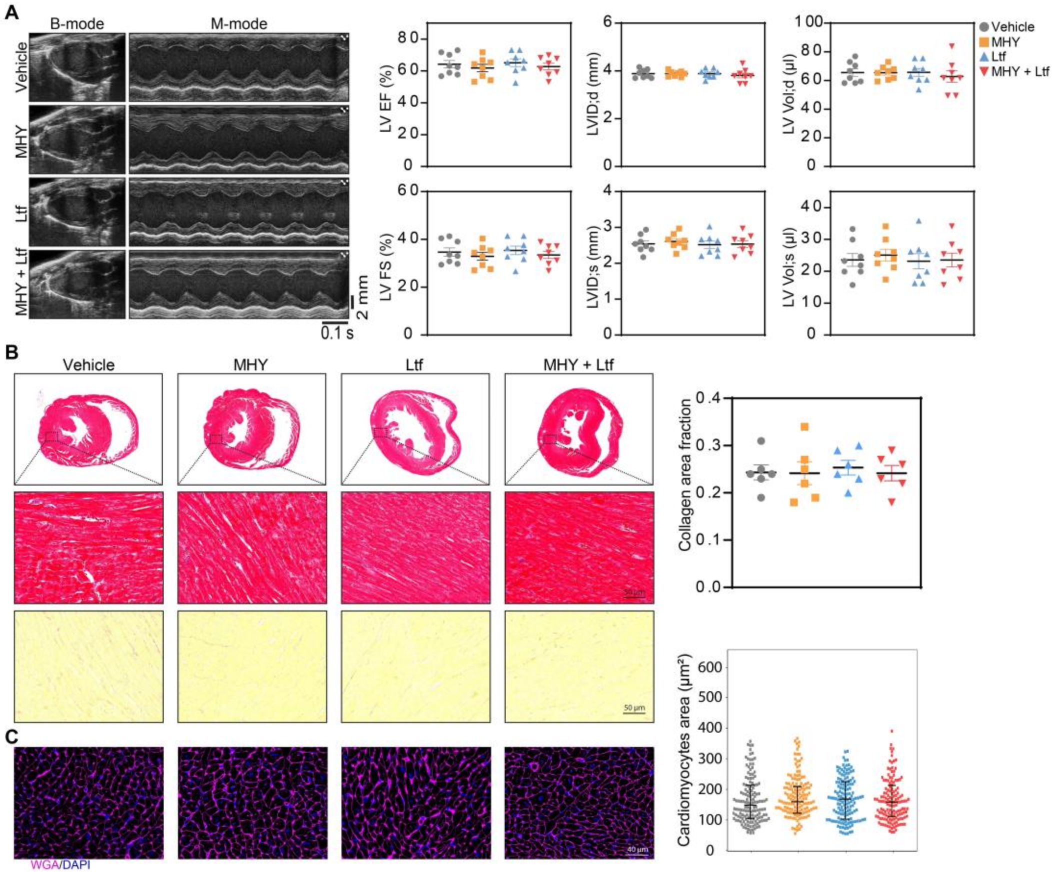

Figure S13. **MHY1485 presents non-cardiotoxic. A** Representative echocardiography and quantifications of relative indicators at 4 weeks after treatment. n = 8/ea. **B**. Representative Masson trichrome-staining and picrosirius red staining images. Quantification of collagen area is presented next to graphs. n = 6/ea. **C**. Representative WGA staining image and quantification. n = 5/ea. Data are presented as mean ± SEM. **A** and **B**, by one-way ANOVA followed by Bonferroni post hoc test; **C**, by Mann–Whitney non-parametric test.

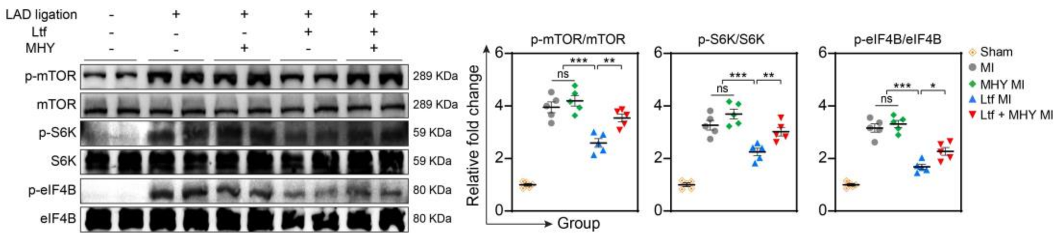

Figure S14. **MHY1485 upregulates activity of mTORC1/S6K/eIF-4B axis suppressed by Ltf.** Representative images of Western blot and quantifications of indicated proteins. n = 5-6/ea. Data are presented as mean ± SEM, by one-way ANOVA followed by Bonferroni post hoc test; *P < 0.05, **P < 0.001, ***P < 0.001 compared with corresponding group.

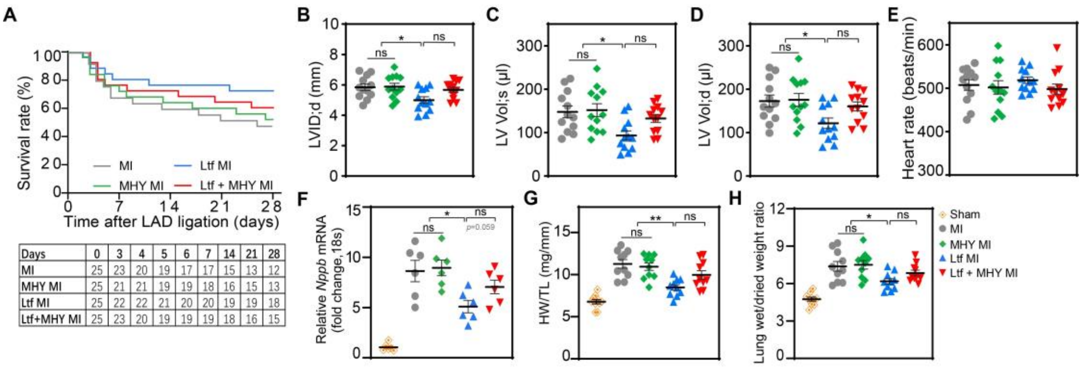

Days	0	3	4	5	6	7	14	21	28
MI	25	23	20	19	17	17	15	13	12
MHY MI	25	21	21	19	19	18	16	15	13
Ltf MI	25	22	22	21	20	20	19	19	18
Ltf+MHY MI	25	23	20	19	19	19	18	16	15

Figure S15. **MHY1485 worsens cardiac outcomes after MI. A**. Survival curve (up) and Survival status (down) of the corresponding subgroups. **B-E**. Quantifications of echocardiographic indicators. n = 12/ea. **F**. Relative *Nppb* mRNA expression level in heart tissue. **G**. Heart weight–to–tibia length ratio (HW/TL). n = 10/ea. **H**. Wet lung weight–to–dried lung weight ratio. n = 10/ea. Data are presented as mean ± SEM. **A**, by Kaplan–Meier survival test; **B-H**, by one-way ANOVA followed by Bonferroni post hoc test; *P < 0.05, **P < 0.001, ***P < 0.001 compared with corresponding group.

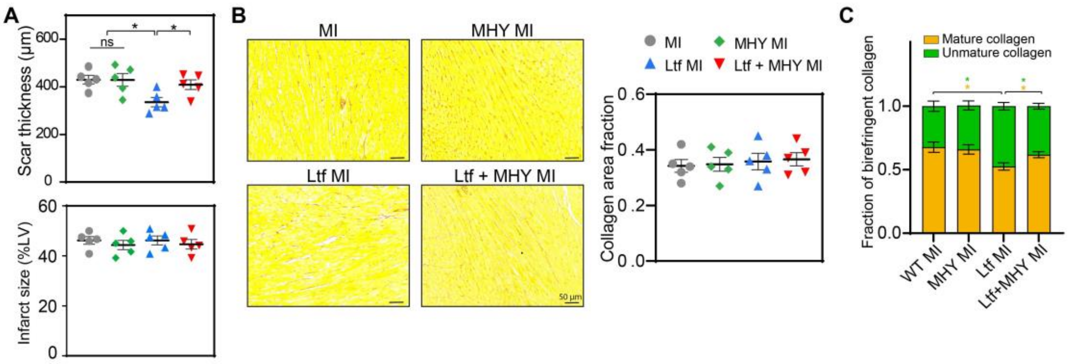

Figure S16. A. Quantifications of scar thickness (up) and infarct size (down) according to pipeline analysis. n = 5/ea. **B**. Representative micrographs (left) and quantification (right) of picrosirius red staining in remote uninjured myocardium. n = 5/ea. **C**. Calculating the frequency of birefringent collagen according to polarized light analysis. n = 5/ea. Data are presented as mean ± SEM, by one-way ANOVA followed by Bonferroni post hoc test, *P < 0.05 compared with corresponding group.

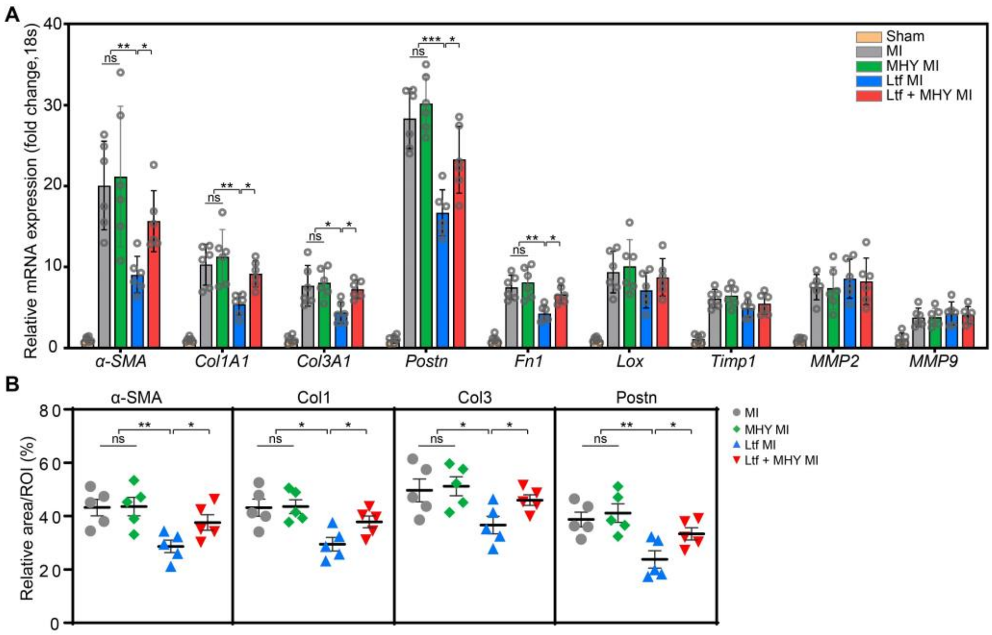

Figure S17. **The effect of Ltf on repressing fibrosis is abolished by MHY1485. A**. mRNA expression level of indicated fibrosis-related genes. n = 6/ea. **B**. Quantifications of immunofluorescent assays. n = 5/ea. Data are presented as mean ± SEM, by one-way ANOVA followed by Bonferroni post hoc test; *P < 0.05, **P < 0.001, ***P < 0.001 compared with corresponding group.

Table S1.

Clinical characteristics of patients with coronary heart disease

Number	31
Age (years)	61.16 ± 11.54
Gender (male)	54.8
Prior hypertension (%)	54.8
Diabetes mellitus (%)	45.2
Total cholesterol (mmol/L)	4.58 ± 1.22
LDL (mmol/L)	2.65 ± 1.01
HDL (mmol/L)	1.18 ± 0.34
IFCC (%)	6.72 ± 1.63
Hemoglobin (g/L)	139.9 ± 15.24
Height (cm)	165 ± 9.78
Body weight (kg)	69.66 ± 12.14
BMI (Kg/m²)	25.58 ± 3.91
Lactoferrin(ng/ml)	529.5 ± 124.9
Echocardiographic Study	
Ejection fraction (%)	62.68 ± 4.51
Left ventricular end systolic diameter (mm)	30.13 ± 4.49
Left ventricular end diastolic diameter (mm)	45.61 ± 4.25
Left atrial diameter (mm)	35.74 ± 5.38
Pulmonary hypertension (mmHg)	27.45 ± 4.65

Clinical characteristics of myocardial infarction patients with heart failure

Number	34
Age (years)	66.15 ± 12.3
Gender (male)	64.7
Prior hypertension (%)	64.7
Diabetes mellitus (%)	41.2
NYHA class	2.14 ± 0.89
Total cholesterol (mmol/L)	4.81 ± 2.02
LDL (mmol/L)	2.96 ± 1.64
HDL (mmol/L)	1.03 ± 0.347
IFCC (%)	6.93 ± 1.76
Hemoglobin (g/L)	136.9 ± 20.8
Height (cm)	165.2 ± 7.5
Body weight (kg)	66.89 ± 16.69
BMI (Kg/m²)	24.32 ± 4.78
Lactoferrin(ng/ml)	404 ± 121.4***
Alanine Aminotransferase (U/L)	52.44 ± 37.7
Aspartate Aminotransferase (U/L)	86.91 ± 62.7
Blood urea nitrogen (mmol/L)	10.15 ± 6.726
Serum creatinine (μmol/L)	106.1 ± 68.93
NT-pro BNP (pg/ml)	8220 ± 9274
Echocardiographic Study	
Ejection fraction (%)	43.5 ± 8.3***
Left ventricular end systolic diameter (mm)	38.53 ± 7.8***
Left ventricular end diastolic diameter (mm)	51.56 ± 6.84***
Left atrial diameter (mm)	39.03 ± 6.38*
Pulmonary hypertension (mmHg)	29.06 ± 10.77

Data are presented as mean ± SD or percentage. Gender, Prior hypertension, Diabetes mellitus are compared by Chi-square test; Age, HDL, Hemoglobin, Lactoferrin, by unpaired *t* test; Total cholesterol, LDL, IFCC, Height, Body weigt, BMI and Echocardiographic Study by Mann–Whitney non-parametric test. *P < 0.05, **P < 0.001, ***P < 0.001 compare with coronary heart disease group.

Table S2.

Day post-MI	0d				1d			
Group	sham	Ltf sham	CTR MI	Ltf MI	sham	Ltf sham	CTR MI	Ltf MI
n	10	10	15	15	10	10	15	15
Age (Weeks)	6-8	6-8	6-8	6-8	6-8	6-8	6-8	6-8
long-axis M-mode								
EF (%)	57.31 ± 1.9	58.93 ± 1.9	56.16 ± 0.91	55.8 ± 1.08	56.31 ± 1.22	56.73 ± 1.45	34.55 ± 1.91	35.49 ± 1.61
FS (%)	29.79 ± 1.27	30.96 ± 1.34	29.01 ± 0.61	28.74 ± 0.71	28.93 ± 0.8	29.24 ± 0.95	16.38 ± 1	16.8 ± 0.84
LVID; d (mm)	3.92 ± 0.06	4.03 ± 0.07	4.08 ± 0.07	4 ± 0.06	3.78 ± 0.08	3.79 ± 0.06	4.18 ± 0.08	4.04 ± 0.06
LVID; s (mm)	2.76 ± 0.08	2.78 ± 0.06	2.9 ± 0.06	2.85 ± 0.06	2.69 ± 0.07	2.68 ± 0.05	3.5 ± 0.09	3.37 ± 0.07
LV Vol; d (μL)	68.39 ± 2.35	71.59 ± 3.04	74 ± 3.06	70.45 ± 2.68	61.68 ± 3	61.81 ± 2.39	78.1 ± 3.62	72.08 ± 2.43
LV Vol; s (μL)	29.29 ± 1.79	29.29 ± 1.62	32.52 ± 1.53	31.29 ± 1.59	27.05 ± 1.72	26.7 ± 1.27	51.45 ± 3.41	46.83 ± 2.57
short-axis M-mode								
EF (%)	56.3 ± 1.52	57.86 ± 2.11	55.41 ± 0.96	55.03 ± 1.34	55.5 ± 1.18	56.58 ± 0.78	34.78 ± 1.79	35.54 ± 1.74
FS (%)	29.1 ± 1.03	30.3 ± 1.44	28.52 ± 0.65	28.29 ± 0.87	28.39 ± 0.76	29.09 ± 0.5	16.49 ± 0.95	16.85 ± 0.9
LVID; d (mm)	4.01 ± 0.05	4.12 ± 0.06	4.08 ± 0.06	4.08 ± 0.07	3.77 ± 0.08	3.8 ± 0.08	4.16 ± 0.08	4.07 ± 0.06
LVID; s (mm)	2.84 ± 0.05	2.87 ± 0.08	2.91 ± 0.05	2.92 ± 0.08	2.71 ± 0.08	2.69 ± 0.07	3.48 ± 0.08	3.39 ± 0.07
LV Vol; d (μL)	70.6 ± 2.12	75.15 ± 2.39	73.57 ± 2.43	72.6 ± 3.49	61.34 ± 3.04	62.27 ± 3.04	77.58 ± 3.7	73.27 ± 2.53
LV Vol; s (μL)	30.8 ± 1.32	31.81 ± 2.07	32.82 ± 1.33	32.94 ± 2.1	27.5 ± 1.89	27.13 ± 1.6	50.76 ± 3.11	47.5 ± 2.58

Day post-MI	7d				14d			
Group	sham	Ltf sham	CTR MI	Ltf MI	sham	Ltf sham	CTR MI	Ltf MI
n	10	10	15	15	10	10	15	15
Age (Weeks)	7-9	7-9	7-9	7-9	8-10	8-10	8-10	8-10
long-axis M-mode								
EF (%)	61.13 ± 1.92	60.25 ± 1.73	27.71 ± 2.18	30.9 ± 1.66	61.76 ± 1.66	62.35 ± 1.71	18.92 ± 2.66	27.83 ± 2.35
FS (%)	32.32 ± 1.3	31.75 ± 1.16	13.03 ± 1.07	14.62 ± 0.82	32.78 ± 1.16	33.21 ± 1.23	8.78 ± 1.28	13.18 ± 1.15
LVID; d (mm)	3.81 ± 0.09	3.91 ± 0.08	5.01 ± 0.17	4.92 ± 0.18	3.87 ± 0.09	3.85 ± 0.09	5.59 ± 0.24	5.4 ± 0.23
LVID; s (mm)	2.59 ± 0.11	2.67 ± 0.08	4.38 ± 0.2	4.21 ± 0.19	2.6 ± 0.09	2.57 ± 0.08	5.13 ± 0.28	4.72 ± 0.26
LV Vol; d (μL)	62.74 ± 3.54	66.5 ± 3.01	121.43 ± 9.54	116.67 ± 10.14	65.2 ± 3.6	64.52 ± 3.53	158.69 ± 16.05	146.38 ± 14
LV Vol; s (μL)	24.94 ± 2.48	26.67 ± 2.02	90.33 ± 9.74	82.46 ± 9.1	25.12 ± 2.06	24.36 ± 1.95	133.11 ± 17.34	109.28 ± 13.54
short-axis M-mode								
EF (%)	61.08 ± 2.22	59.81 ± 1.41	25.35 ± 2.6	28.98 ± 1.77	60.98 ± 1.76	61.45 ± 1.72	19.46 ± 2.71	28.66 ± 2.71
FS (%)	32.37 ± 1.59	31.43 ± 0.97	11.88 ± 1.28	13.61 ± 0.86	32.28 ± 1.25	32.63 ± 1.22	9.06 ± 1.3	13.65 ± 1.35
LVID; d (mm)	3.82 ± 0.1	3.96 ± 0.08	5.03 ± 0.17	4.87 ± 0.18	3.91 ± 0.07	3.95 ± 0.08	5.65 ± 0.25	5.46 ± 0.26
LVID; s (mm)	2.59 ± 0.12	2.72 ± 0.08	4.46 ± 0.21	4.22 ± 0.2	2.65 ± 0.08	2.67 ± 0.09	5.17 ± 0.29	4.75 ± 0.29
LV Vol; d (μL)	63.23 ± 3.82	68.71 ± 3.49	122.85 ± 9.93	114.1 ± 10.43	66.52 ± 2.7	68.33 ± 3.53	162.48 ± 16.66	150.96 ± 15.53
LV Vol; s (μL)	25.23 ± 2.73	27.84 ± 2.06	94.74 ± 10.63	83.01 ± 9.67	26.12 ± 1.86	26.65 ± 2.34	135.56 ± 17.63	112.72 ± 15.04

Day post-MI	28d			
Group	sham	Ltf sham	CTR MI	Ltf MI
n	10	10	15	15
Age (Weeks)	10-12	10-12	10-12	10-12
long-axis M-mode				
EF (%)	58.09 ± 1.25	59.41 ± 1.13	15.46 ± 1.93	27.05 ± 1.92
FS (%)	30.24 ± 0.83	31.14 ± 0.8	7.1 ± 0.91	12.75 ± 0.93
LVID; d (mm)	3.97 ± 0.06	3.97 ± 0.08	5.95 ± 0.23	5.42 ± 0.2
LVID; s (mm)	2.77 ± 0.06	2.73 ± 0.05	5.55 ± 0.26	4.75 ± 0.22
LV Vol; d (μL)	68.87 ± 2.43	68.93 ± 3.15	181.77 ± 16.17	146.39 ± 12.35
LV Vol; s (μL)	29 ± 1.59	27.9 ± 1.28	156.71 ± 16.3	109.56 ± 11.96
short-axis M-mode				
EF (%)	57.74 ± 1.1	59.14 ± 1.24	14.72 ± 1.54	26.09 ± 2.06
FS (%)	30.02 ± 0.74	31.01 ± 0.86	6.72 ± 0.72	12.28 ± 1.02
LVID; d (mm)	4 ± 0.06	4.03 ± 0.09	5.96 ± 0.24	5.44 ± 0.21
LVID; s (mm)	2.8 ± 0.05	2.78 ± 0.06	5.57 ± 0.26	4.8 ± 0.23
LV Vol; d (μL)	70.21 ± 2.64	71.73 ± 3.52	182.59 ± 16.58	148.41 ± 13.72
LV Vol; s (μL)	29.7 ± 1.42	29.18 ± 1.41	158.06 ± 16.18	112.68 ± 13.26

Data are presented as mean ± SEM.

Table S3.

Group	WT MI	MHY MI	Ltf MI	Ltf+MHY MI
n	12	12	12	12
Age (Weeks)	10-12	10-12	10-12	10-12
EF (%)	14.92 ± 1.32	14.25 ± 1.63	23.86 ± 1.97	17.61 ± 1.4
FS (%)	6.83 ± 0.63	6.5 ± 0.77	11.06 ± 0.97	8.08 ± 0.67
LVID; d (mm)	5.84 ± 0.21	5.88 ± 0.22	5 ± 0.22	5.67 ± 0.16
LVID; s (mm)	5.45 ± 0.22	5.51 ± 0.23	4.45 ± 0.21	5.22 ± 0.16
LV Vol; d (μL)	172.6 ± 14.16	175.6 ± 14.85	121.6 ± 11.97	160.3 ± 10.26
LV Vol; s (μL)	148 ± 13.66	151.9 ± 14.71	93.46 ± 10.54	132.5 ± 9.25
Heart rate (beats/min)	507.3 ± 12.13	501.7 ± 15.53	518.5 ± 7.56	498 ± 11.66

Data are presented as mean ± SEM

Table S4. Primers for RT-PCR.

Gene	Forward Primer (5' to 3')	Reverse Primer (5' to 3')
Lactoferrin	CCGCCGCTCAGTTGTG	CAGGTCGCAGTTTGTAGGG
Nppb	GCACAAGATAGACCGGATCG	CCCAGGCAGAGTCAGAAAC
Nppa	GGCTTCTTCCTCGTCTTGG	CAGGTGGTCTAGCAGGTTCT
Col3a1	CTGTAACATGGAAACTGGGGAAA	CCATAGCTGAACTGAAAACCACC
Col1a1	TCGTGGCTTCTCTGGTCTC	CCGTTGAGTCCGTCTTTGC
Postn	CGGGAAGAACGAATCATTACA	ACCTTGGAGACCTCTTTTTGC
Vimentin	ACTAGCCGCAGCCTCTATTCCTC	GAAGTCCACCGAGTCTTGAAGCAG
αSMA	GTCCCAGACATCAGGGAGTAA	TCGGATACTTCAGCGTCAGGA
Fn1	AAGAGGACGTTGCAGAGCTA	AGACACTGGAGACACTGACTAA
Timp1	GCATCTGGCATCCTCTTGTTG	GGTGGTCTCGTTGATTTCTGG
MMP2	TGTCTTGCGTCTGACACTGC	CTCCTTTGGGCTAGGTATCTCT
MMP9	AGACGACATAGACGGCATCC	TGGGACACATAGTGGGAGGT
Lox	GACATTCGCTACACAGGACAT	AACACCAGGTACGGCTTTATC
18s	ACCGCAGCTAGGAATAATGGA	CAAATGCTTTCGCTCTGGTC

Table S5. Antibodies used in this study.

Antibody	Company	Cat.No.	Application	Dilution
Lactoferrin	Invitrogen	PA5-95513	WB	1:1000
Lactoferrin	Invitrogen	PA5-95513	IP	1:100
Collagen 1	Abcam	ab34710	WB	1:1000
Collagen 1	Abcam	ab34710	IF	1:200
Collagen 3	Abcam	ab7778	WB	1:1000
Collagen 3	Abcam	ab7778	IF	1:200
α-SMA	Abcam	ab7817	WB	1:1000
α-SMA	Abcam	ab7817	IF	1:200
Vimentin	Abcam	ab92547	WB	1:1000
Postn	Proteintech	66491-1-Ig	WB	1:2000
Postn	Proteintech	66491-1-Ig	IF	1:1000
MMP2	Abcam	ab92536	IF	1:200
MMP9	Abcam	ab76003	IF	1:200
mTOR	Abcam	ab134903	WB	1:1000
p-mTOR	Abcam	ab109268	WB	1:1000
S6k	Abcam	ab32359	WB	1:1000
p-S6k	Abcam	ab60948	WB	1:1000
eIF4B	Proteintech	17917-1-AP	WB	1:1000
p-eIF4B	Abcam	ab59300	WB	1:1000
AKT	CST	9272	WB	1:1000
p-AKT	CST	4060	WB	1:1000
CD74	Abcam	ab289885	WB	1:1000
CD74	Abcam	ab289885	IP	1:30
CD74	Proteintech	66390-1-Ig	IF	1:400
Ki67	Abcam	ab15580	IF	1:200
β-actin	Proteintech	HRP-60008	WB	1:20000
